# Vinylation of a Secondary Amine Core with Calcium Carbide for Efficient Post-Modification and Access to Polymeric Materials

**DOI:** 10.3390/molecules23030648

**Published:** 2018-03-13

**Authors:** Konstantin S. Rodygin, Alexander S. Bogachenkov, Valentine P. Ananikov

**Affiliations:** 1 Saint Petersburg State University, Universitetsky Prospect, 26, St. Petersburg 198504, Russia; konstantinrs@rambler.ru (K.S.R.); alexterve@gmail.com (A.S.B.); 2 Zelinsky Institute of Organic Chemistry, Russian Academy of Science, Leninsky Prospect, 47, Moscow 119991, Russia

**Keywords:** acetylene, calcium carbide, vinylation, olanzapine, functionalization

## Abstract

We developed a simple and efficient strategy to access *N*-vinyl secondary amines of various naturally occurring materials using readily available solid acetylene reagents (calcium carbide, KF, and KOH). Pyrrole, pyrazole, indoles, carbazoles, and diarylamines were successfully vinylated in good yields. Cross-linked and linear polymers were synthesized from *N*-vinyl carbazoles through free radical and cationic polymerization. Post-modification of olanzapine (an antipsychotic drug substance) was successfully performed.

## 1. Introduction

Nitrogen-containing compounds are one of the most important functional molecules in modern chemistry, biology, and drug development. Nucleic acids, *N*-heterocyclic compounds, amines, amino-acids, and their biopolymers are pivotal to a vast majority of biochemical processes. Effective modification of secondary amines by the insertion of a vinyl group leads to demanded *N*-vinyl derivatives with a variety of new applications. Vinylated products are interesting both as biologically active cores [1] and as versatile building blocks for polymerization [2]. At the same time, carbazole-based compounds are considered promising as photoconductors and charge-transporting materials [3] in the aftermath of the discovery of photoconductivity in poly(*N*-vinylcarbazole) [4]. The unique properties of vinyl amines are exemplified by their ability to polymerize under both cationic [5] and free radical conditions [6]. Polymer synthesis with *N*-heterocyclic moieties like diazepine has great potential for application. The biologically active core can be converted into a polymeric form, which is capable of gradual depolymerization and release under specific conditions.

**Scheme 1:**
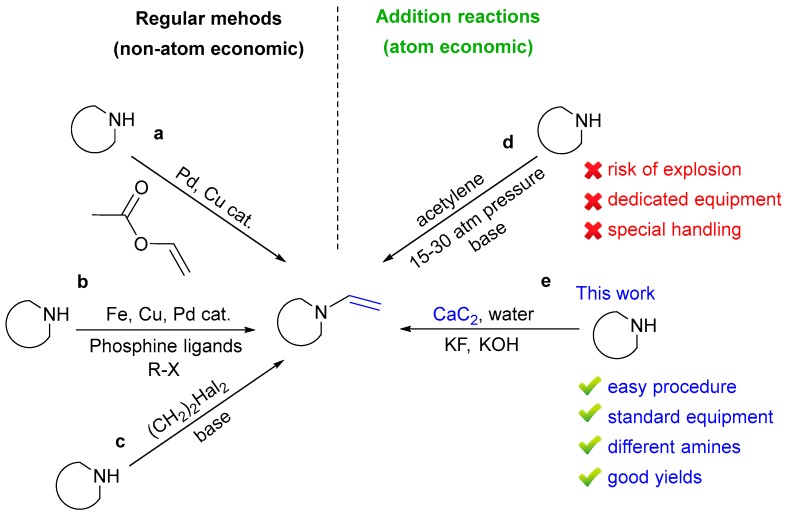
Synthetic procedures to access *N*-vinyl derivatives.

Current methods for preparing *N*-vinyl derivatives of secondary amines involve the vinyl exchange reaction (Scheme 1, route a), which requires a source of vinyl group (in most cases represented by vinyl acetate) and a metal catalyst. Enamines from nitrogenous bases [7], imidazole [8], triazoles [9], carbazole [10], and pyrrolidone [11] can be successfully obtained by this approach.

Cross-coupling reactions, catalyzed by copper [12] and iron [13], or palladium complexes [14], require a partner: trimethyloxyvinylsilane [15], potassium vinyltrifluoroborate [16], or vinyl halide [17] (Scheme 1, route b). Another non-atom economic strategy, elimination, is based on a two-step sequence, which is carried out under basic conditions (Scheme 1, route c). The Clemo-Perkin method [18] and dehydrohalogenation in the presence of phase transfer catalyst [19] lead to *N*-vinyl derivatives of indoles [20], carbazoles [21], imidazoles [22], pyrazoles, triazoles, or tetrazoles [23] and nitrogenous bases [24]. Several other transformations leading to *N*-vinyl derivatives, such as dehydrogenation [25], ethylene insertion [26], and amine/aldehyde condensation could be also mentioned [27].

An excellent atom-economic opportunity is provided by the addition reaction of acetylene, proceeding without by-product formation, which is to say that all atoms of starting materials become incorporated into products. This reaction (Scheme 1, route d) was originally implemented by Reppe [28], and it is still used with several optimizations [29]. Arylamines can be successfully vinylated according to this procedure [30]. However, using and handling pressured acetylene is technically difficult and requires dedicated hardware [31,32]. Not surprisingly, this atom-economic approach, despite being efficient in terms of chemistry, has not found widespread application in synthetic practice.

As an alternative to high pressure acetylene, calcium carbide has demonstrated a very impressive potential in organic transformations [32,33,34,35,36,37,38,39,40]. Calcium carbide is inexpensive and readily available at the large-scale commercial production level. Currently, a carbide-based synthesis is underestimated in its potential to provide not only useful intermediates for lab chemistry, but also materials and polymers for fine technology [32,40]. As representative examples, the vinylation of O-H and S-H groups has been reported [36,37,38,39,40]. However, the vinylation of the N-H group is challenging, and only indole derivatives have been studied [41]. Recently, we developed a concept involving a solid acetylene reagent, where the reactivity of calcium carbide and in situ generated acetylene was tuned by the addition of KF [38]. 

In this work, we developed a synthetic methodology for preparing *N*-vinyl derivatives of secondary amines by utilizing a solid acetylene reagent (Scheme 1, route e). Fluoride-mediated vinylation is a powerful and new approach not addressed previously for the functionalization of amines. The vinylation of pyrrole, pyrazole, carbazoles, and diarylamines is reported here for the first time. This method has a number of key advantages from the standpoint of green chemistry (Scheme 1, route e) and a very simple practical procedure was developed using a combination of CaC_2_/KOH/KF.

## 2. Results and Discussion

Optimization of the proposed system was carried out to find the optimal conditions, particularly the solvent and the temperature. First, a dedicated experiment was performed to choose a solvent: pieces of calcium carbide were exposed to a solvent/water mixture under stirring. In cases of hydrophobic solvents (e.g., hexane or toluene), two phases appeared, and calcium carbide reacted much faster when the water was heavier than the solvent. In both cases, the release of acetylene did not match the rate of nucleophilic addition to the triple bond, and the product yields were low (Table 1, entries 1 and 2). In cases of dimethyl sulfoxide (DMSO) and dimethyl formamide (DMF), a sufficiently slow release of gaseous acetylene allowed us to obtain and utilize its constant flow. To minimize losses, we finally chose to employ DMSO, avoiding DMF for its partial decomposition during the reaction. Furthermore, acetylene has good solubility in DMSO, and inorganic salts are also soluble in DMSO.

Reaction in DMSO gave the product in 38% yield (Table 1, entry 3), which was further improved to 80% (Table 1, entry 4). Increasing loads of CaC_2_ and water did not improve the yields (Table 1, entries 5, 6). Different bases were probed (NaOH, TEA, K_2_CO_3_, Na_2_CO_3_, pyridine), and potassium hydroxide turned out to be the most suitable. It was also important to use both components—KOH and KF—simultaneously, since using them separately significantly decreased the yields (Table 1, cf. entries 7, 8, and 4). Interesting to note, the usage of KF provided a higher yield as compared to KOH (Table 1, entries 7, 8).

Performing sequential vinylation by splitting the amount of calcium carbide into two parts provided an excellent product yield of 88% (Table 1, entry 9). The optimum temperature was estimated: lower temperatures led to incomplete conversion (Table 1, entry 10), while higher temperatures resulted in the decomposition and polymerization of the product (Table 1, entry 11). The optimal amount of base was also established; further increasing its amount led to a decrease in the product yields (Table 1, entry 12). The presence of water was necessary, as indicated by the control experiment, in which the reaction did not start without water in dry DMSO (Table 1, entry 13).

**Scheme 2:**
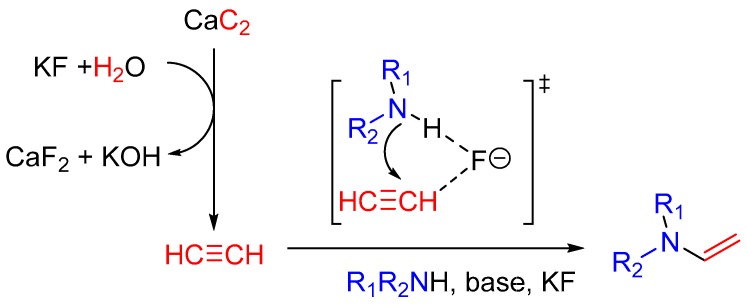
Plausible mechanism of vinylation of secondary amines.

The role of potassium fluoride in the studied system represents an interesting question. Microscopic examination of the solid inorganic postreaction waste, obtained in the absence of KF, indicated the formation of Ca(OH)_2_, as could be expected due to reaction of calcium carbide with water. The presence of KF promoted the formation of CaF_2_ as a predominant component of the inorganic postreaction residue. The observed difference is explained by the lower solubility and higher stability of CaF_2_ [38]. The presence of potassium fluoride in the developed system importantly contributes to the reactivity of calcium carbide and allows the controlled release of acetylene (Scheme 2). Another important role may include the fluoride-mediated functionalization of acetylene (Scheme 2). A similar effect was reported for the concept of a solid acetylene reagent in the OH bond vinylation process [38].

**Scheme 3:**
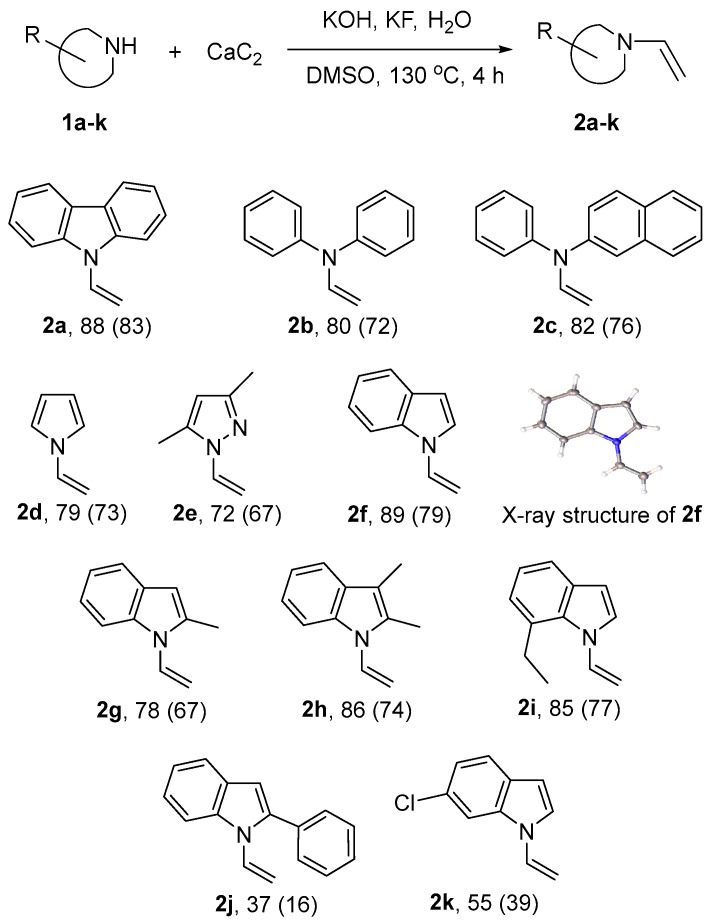
Structures of vinylated secondary amine products and yields (in %). Reaction conditions: amine **1** (1 mmol), CaC_2_ (2 mmol), KOH (1.1 mmol), KF (1 mmol), H_2_O (4 mmol), DMSO (1 mL). NMR yields are given without parentheses, isolated yields are given in parentheses.

**Scheme 4:**
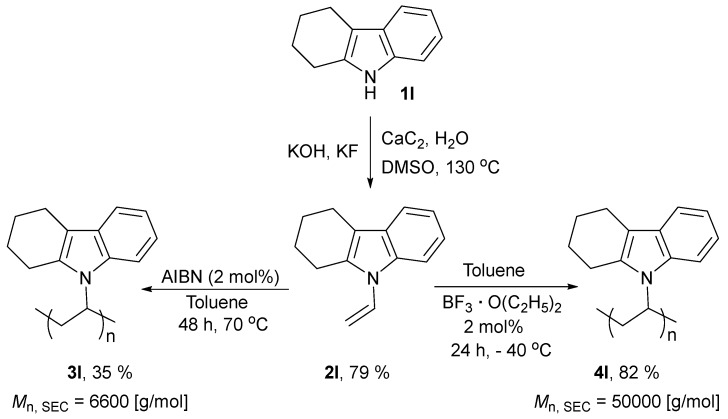
*N*-vinyl-1,2,3,4-tetrahydrocarbazole synthesis and polymerization.

**Scheme 5:**
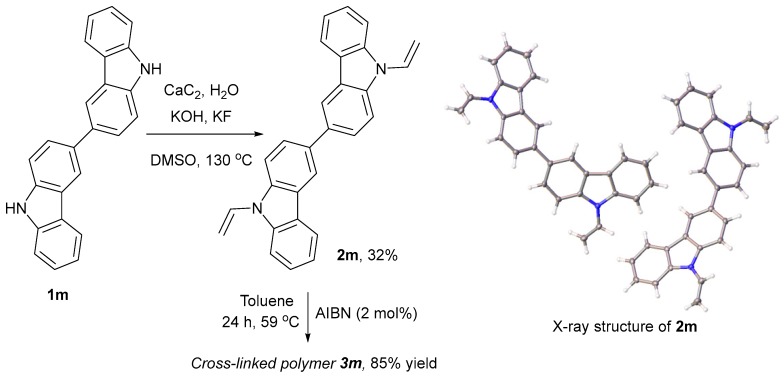
Double vinylation followed by the polymerization of bis(*N*-vinyl-3,3′-carbazole) and the X-ray structure of **2m**.

To assess the substrate scope of the developed vinylation protocol, it was applied to arylamines (**2b**, **2c**), pyrrole (**2d**), pyrazole (**2e**), indoles (**2f**–**k**), tetrahydrocarbazole (**2l**), bicarbazole (**2m**), and diazepine (**2n**) (Scheme 3, Scheme 4 and Scheme 5). The vinylation of aryl amines was flawless and produced excellent yields (**2b**, **2c**). Pyrrole (**2d**) and pyrazole (**2e**) were also successfully vinylated. The vinylation of indoles delivered moderate to good product yields (**2f**–**k**). Surprisingly, despite *N*-vinylindole being a well-known compound [42], its X-ray structure is considered obscure due to its low melting point (about 30 °C) [43]. Extraction with hexane applied in this study allowed the isolation of pure vinylated derivatives, which included *N*-vinylindole, and the determination their structures by means of X-ray crystallography (Scheme 3).

The yield of vinyl indoles depended on the nature of a substituent in the aromatic ring. Substituents with an electron-withdrawing effect, especially in the 2-position, substantially decreased the yield (**2j**). This effect was probably due to the delocalization of negative charge throughout the molecule after the proton elimination and corresponding decrease in reactivity of the nucleophile. Isolated vinylindoles were air- and moisture-sensitive, especially in presence of trace amounts of acids. Devinylation occurred as hydrolysis to an initial indole and acetic aldehyde. Simplicity of the synthetic procedure is noteworthy; the products can be thoroughly purified through extraction with hexane (other postreaction components are insoluble inorganic molecules).

Upon the extraction of the vinylated carbazoles (**2a**, **2l**), their low solubility in nonpolar solvents became evident, and hexane was replaced by ether for more efficient separation.

It was very interesting to probe the vinylated tetrahydrocarbazole (**2l**) in polymerization reactions (Scheme 4). To try the possibility of polymerization, we used *N*-vinyl-1,2,3,4-tetrahydrocarbazole (**1l**) as a monomer in either free radical or cationic conditions (Scheme 4). The **3l** oligomer was isolated in low yield and moderate average mass after free radical polymerization in the presence of AIBN (2 mol %) in toluene medium. The electron-donating and steric effects of methylene groups led to a decreased molecular mass of the **3l** polymer as compared with polymers derived from completely aromatic carbazole. The presence of such substituents in carbazoles also led to decreasing molecular weight. Cationic polymerization worked better, leading to a polymer of a greater mass (**4l**). Note that a simple protocol was utilized and reaction conditions were not optimized. Both polymers were reprecipitated from methanol and characterized by NMR and size exclusion chromatography (SEC). 

Comparing monomers **2a** and **2l**, one may note that both *N*-vinyl derivatives easily undergo cationic polymerization and produce polymers of similar molecular mass range. However, different behavior was observed in the case of free radical polymerization, where the vinylated tetrahydrocarbazole (**2l**) gave shorter polymer chains. Thus, access to both monomers provides good potential for material science applications as an extension of well-known carbazole-containing systems.

3,3′-bicarbazole (**1m**) was chosen as a model substrate for further polymerization experiments in connection with the promising potential of polyvinyl carbazole (PVC) in optoelectronics. One-pot insertion of two vinyl groups is challenging, since spontaneous oligomerization may readily occur during the synthetic procedure. Nevertheless, the individual double vinylated 3,3′-bicarbazole (**2m**) was isolated in 32% yield using the developed procedure with calcium carbide (Scheme 5). The presence of two vinyl groups in the monomer was clearly confirmed by X-ray analysis (Scheme 5).

Polymerization of the bis-vinyl derivative in the presence of radical initiator (AIBN) resulted in high yields (85%) of the insoluble cross-linked polymer **3m**. Comparison of the registered solid-state NMR-spectra with literature-derived NMR data on PVC [44] indicated the polymeric nature of the obtained material (see Supplementary Materials for details). 

**Scheme 6:**
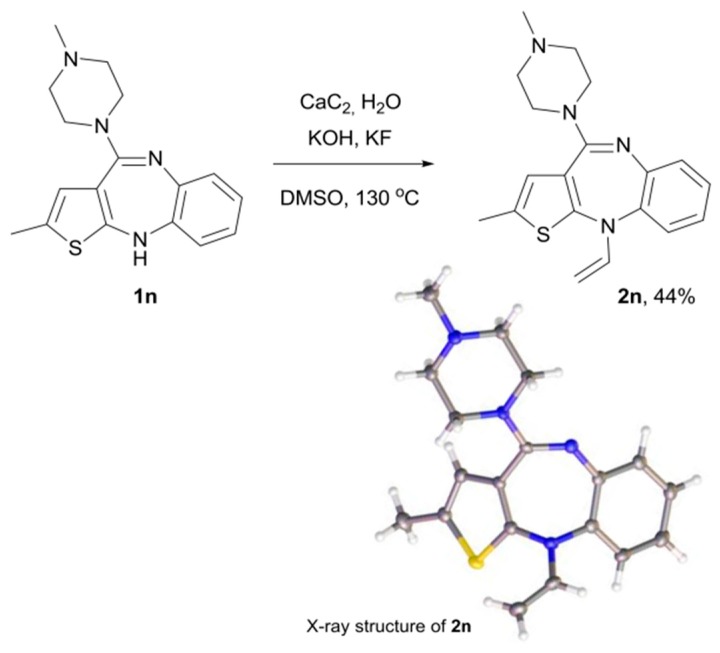
Vinylation of olanzapine and X-ray structure of the vinylated olanzapine derivative.

The developed vinylation procedure was successfully utilized for post-modification of a drug substance. Olanzapine (Zyprexa) is thienobenzodiazepine used for the treatment of schizophrenia and bipolar disorder [45]; it acts by suppressing dopamine and serotonin receptor activities. Currently available antipsychotic drugs have certain limitations. Considerable academic efforts are aimed at enhancing their properties (e.g., by chemical post-modification), including the development of new platforms for drug delivery [46]. The insertion of vinyl groups modulates the functionality of a biologically active benzodiazepine core and creates novel opportunities for molecular binding. An olanzapine-containing polymer, slowly releasing the active units by gradual depolymerization and/or devinylation upon delivery, could be of great therapeutic relevance. The vinylation of olanzapine (**1n**) under the developed conditions proceeded smoothly (Scheme 5), and the vinyl derivative (**2n**) was isolated in a pure form as a solid substance. The molecular structure of product **2n** was determined by X-ray analysis (Scheme 6).

## 3. Materials and Methods

Granulated calcium carbide was purchased from Sigma Aldrich (St. Louis, MO, USA) (≥75% volumetric). NMR spectra of the compounds were recorded using a Bruker Avance DRX 400 spectrometer at 298 K. The ^1^H and ^13^C-NMR chemical shifts are reported in ppm and were determined by referencing the peaks to the residual solvent signals. The data were processed using MestReNova (version 6.0.2) desktop NMR data processing software. High-resolution mass spectra were registered on a Bruker Micro-TOF mass spectrometer (ESI-MS). All melting points (m.p.) were measured in open capillaries on an electrothermal apparatus and are uncorrected. Liquid monomers were distilled over calcium hydride in vacuum before polymerization. Size exclusion chromatography (SEC) was carried out using a Shimadzu LC-20AD modular system equipped with a TSKgel G5000HHR column (7.8 mm × 300 mm) and an RID-10A differential refractive index detector. The average molar mass (${\bar{M}n}_{\mathrm{SEC}}$) and molar mass distribution ($\bar{M}w$/$\bar{M}n$) values were determined using SEC in tetrahydrofurane (THF) at 40 °C (flow rate = 1.0 mL·min^−1^) vs. polystyrene standards. The unit calibration was conducted using commercially available narrow molecular-weight-distribution polystyrene standards (0.5–1000 kDa, Polymer Laboratories). The chromatograms were processed using Shimadzu LCsolution software. The polymer samples were initially filtered through a pulytetrafluoroethylene (PTFE) filter (0.45 µm, 13 mm, Macherey-Nagel) and dissolved in THF (6 mg·mL^−1^). Purification via column chromatography on silica should be avoided due to the sensitivity of most of the *N*-vinyl amines towards undergoing rapid polymerization or degradation. If purification via column chromatography is required for some reason, the silica should be neutralized with triethylamine. The products may be sensitive to light and should be stored in a dark place. Contact with acid or traces of metals may initiate polymerization and should be avoided.

### 3.1. Synthetic Procedures

#### 3.1.1. General Procedure of Vinylation

An amine (1.0 mmol), crushed KOH (1.1 mmol, 62 mg), anhydrous KF (1.0 mmol, 58 mg), and granulated calcium carbide (2.0 mmol, 130 mg) were added to a reaction tube (7 mL) with 1 mL of DMSO. After stirring at room temperature for 5 min, water (4.0 mmol, 72 μL) was added, the tube was sealed, and the mixture was heated at 130 °C for 4 h with vigorous stirring. After cooling to 25 °C, the mixture was extracted with hexane (4 × 4 mL). Combined extracts were treated with 5% aqueous NaOH, then with brine, with water, and finally dried over Na_2_SO_4_. Concentration under reduced pressure gave the target compound. All vinylated amines were obtained as oils except 9-vinyl-9*H*-carbazole (**2a**) (m.p. 64–65 °C, lit. 64 °C [42]), *N*,*N*-diphenylvinylamine (**2b**) (m.p. 53–54 °C, lit. 52–54 °C [47]), *N*-(β-naphthyl)-*N*-phenylvinylamine (**2c**) (m.p. 80–81 °C, lit. 70–82 °C [47]), 9,9′-divinyl-9*H*,9′*H*-3,3′-bicarbazole (**2m**) (decomposes at 162 °C), and 2-methyl-4-(4-methylpiperazin-1-yl)-10-vinyl-10*H*-benzo[b]thieno[2,3-e][1,4]diazepine (**2n**) (m.p. 188–190 °C).

In case of compounds **2m** and **2n**, methyl *tert*-butyl ether (MTBE) was used for extraction instead of hexane. *N*-vinyl derivatives were isolated by flash column chromatography with system hexane/MTBE (5/1) as an eluent with gradient elution. Compounds characterization is reported in the Supplementary Materials.

Caution: The experimental procedures described in the present study involve the evolution of gaseous acetylene upon the reaction of water with calcium carbide—the necessary safety requirements for experiments with gases, acetylene, and CaC_2_ should be implemented (see corresponding regulations).

#### 3.1.2. Experimental Procedure for the Radical Polymerization of *N*-vinyl-1,2,3,4-tetrahydrocarbazole (**1l**)

To start, 276 mg (1.4 mmol) of **1l** and 5 mg (0.03 mmol) of AIBN were added to a Schlenk tube containing 1 mL of dry and degassed toluene. The tube was sealed, and the reaction was performed with stirring under an inert atmosphere at 70 °C for 48 h. Then, the reaction mixture was precipitated in methanol (20 mL). The crude product was dissolved in chloroform (1 mL) and again precipitated in methanol (20 mL). After the residue was washed with hexane (3 × 3 mL), the work-up procedure was repeated. The residue was dried under a reduced pressure for two days at 40 °C to obtain a yellow solid (97 mg, 35% yield, *M*_n,SEC_ = 6600 (g/mol), *Đ* = 1.50).

#### 3.1.3. Experimental Procedure for the Cationic Polymerization of *N*-vinyl-1,2,3,4-tetrahydrocarbazole (**1l**)

To start, 276 mg (1.4 mmol) of **1l** was placed into a Schlenk tube under an inert atmosphere. Then, 1 mL of degassed dry toluene was injected into the tube. After three degassing cycles, a toluene solution of boron trifluoride diethyl etherate (2 mol %) was injected into the tube under stirring at −40 °C. After 24 h in a cryostat, the toluene solution was poured into methanol. The yellow solid was collected through a filter, washed with hexane, dissolved in chloroform, and precipitated in methanol again. The powder was dried under a reduced pressure for two days at 40 °C (226 mg, 82% yield, *M*_n,SEC_ = 50000 (g/mol), *Đ* = 1.61).

## 4. Conclusions

In conclusion, a new strategy of vinylation was developed, and a variety of secondary amines were effectively converted into *N*-vinyl derivatives. Calcium carbide was utilized as a vinylation agent to avoid high pressure equipment when producing required amounts of acetylene and thereby simplify the synthetic procedure. The application of KF/KOH as a base was important for efficient transformation. The reaction turned out to have a good substrate scope, and various pyrrole, pyrazole, indoles, aryl amines, and carbazoles were successfully involved in the transformation. Regular and cross-linked polymers were obtained by radical and cationic polymerization of the studied carbazole- and bicarbazole-containing substrates. *N*-vinyl olanzapine was synthesized, and its molecular structure was established for the first time.

## Figures and Tables

**Table 1:**
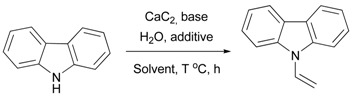
Optimization of the reaction conditions. ^1^

Entry	Solvent	Base + Additive, equiv.	CaC_2_, equiv.	Water, equiv.	T, °C	Time, h	Yield, % ^2^
1	Hexane	KOH (1.1), KF (1)	2	4	130	4	trace
2	Toluene	KOH (1.1), KF (1)	2	4	130	4	trace
3	DMSO	KOH (1.1), KF (1)	2	4	130	8	38
4 ^3^	DMSO	KOH (1.1), KF (1)	2	4	130	4	80
5	DMSO	KOH (1.1), KF (1)	2	8	130	4	62
6	DMSO	KOH (1.1), KF (1)	4	8	130	4	57
7	DMSO	KF (1)	2	4	130	4	54
8	DMSO	KOH (1.1)	2	4	130	4	25
9 ^4^	DMSO	KOH (1.1), KF (1)	2	4	130	4	88
10	DMSO	KOH (1.1), KF (1)	2	4	110	4	66
11	DMSO	KOH (1.1), KF (1)	2	4	150	4	50
12	DMSO	KOH (1.5), KF (1.5)	2	4	130	4	52
13 ^5^	DMSO	KOH, KF	2	-	130	4	trace

^1^ Reaction conditions: substrate (1 equiv.), CaC_2_ (2 equiv.), KOH (1.1 equiv.), KF (1 equiv.), H_2_O (4 equiv.), DMSO (1 mL), 4 h; ^2^ NMR yields; ^3^ DMSO 3 mL; ^4^ the yield after double vinylation, the yield after single vinylation was 84%; ^5^ dry DMSO.

## Supplementary Materials

Supplementary data are available online.
