# Supplementary material for: Vinylation of a Secondary Amine Core with Calcium Carbide for Efficient Post-Modification and Access to Polymeric Materials
Source: Molecules. 2018 Mar 13;23(3):648. doi: 10.3390/molecules23030648 (PMC6017323; doi:10.3390/molecules23030648)
Supplement: Supplementary file 1 [file molecules-23-00648-s001.zip › Supporting-Information-corrected.pdf]

Supporting Information for the Article

Vinylation of a Secondary Amine Core with Calcium Carbide for Efficient Post-Modification and Access to Polymeric Materials

Konstantin S. Rodygin,[a] Alexander S. Bogachenkov,[a] Valentine P. Ananikov[a,b] *

[a] *Saint Petersburg State University, Universitetskii prospect, 26, Petergof, Russia.*

[b] *N.D. Zelinsky Institute of Organic Chemistry, Russian Academy of Sciences, Leninsky Prospect, 47, Moscow, Russia;. e-mail: val@ioc.ac.ru*

Content

Characterization and spectral data 2

NMR spectra 6

Crystal structures 21

Characterization and spectral data

9-Vinyl-9*H*-carbazole (2a)

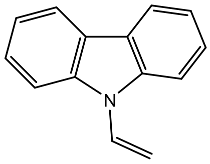

Yield, %: 88 (by NMR); 83 (isolated). **^{1}H NMR** (400 MHz, DMSO-d_6) δ 8.19 (d, *J* = 8.0 Hz, 2H), 7.85 (d, *J* = 8.0 Hz, 2H), 7.60 (dd, *J* = 16.0, 8.0 Hz, 1H), 7.50 (t, *J* = 8.0 Hz, 2H), 7.31 (t, *J* = 8.0 Hz, 2H), 5.61 (d, *J* = 16.0 Hz, 1H), 5.13 (d, *J* = 8.0 Hz, 1H); **^{13}C NMR** (101 MHz, DMSO-d_6) δ 138.7, 129. 8, 126.5, 123.3, 120.7, 120.3, 110.9, 101.1; **HRMS** (m/z): $[M+H]^+$ calcd. for $C_{14}H_{11}NH^+$, 194.0964; found, 194.0971.

N,N-Diphenylvinylamine (2b)

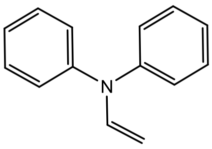

Yield, %: 80 (by NMR); 72 (isolated). **^{1}H NMR** (400 MHz, acetone-d_6) δ 7.38−7.33 (m, 4H), 7.13−7.09 (m, 2H), 7.05−6.97 (m, 5H), 4.10 (d, *J* = 8.0 Hz, 1H), 3.92 (d, *J* = 12.0 Hz, 1H); **^{13}C NMR** (101 MHz, acetone-d_6) δ 146.1, 138.9, 130.4, 124.5, 124.2, 90.5; **HRMS** (m/z): $[M+H]^+$ calcd. for $C_{14}H_{13}NH^+$, 196.1121; found, 196.1118.

N-(β-Naphthyl)-N-phenylvinylamine (2c)

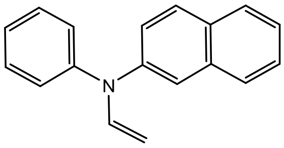

Yield, %: 82 (by NMR); 76 (isolated). **^{1}H NMR** (400 MHz, DMSO-d_6) δ 7.86−7.82 (m, 2H), 7.78 (d, *J* = 8.0 Hz, 1H), 7.49−7.35 (m, 5H), 7.16−7.05 (m, 5H), 4.18 (d, *J* = 8.0 Hz, 1H), 3.93 (d, *J* = 16.0 Hz, 1H); **^{13}C NMR** (101 MHz, DMSO-d_6) δ 144.4, 142.2, 138.1, 134.0, 129.9, 129.7, 129.2, 127.5, 127.1, 126.5, 124.8, 124.0, 123.6, 122.9, 119.0, 90.8; **HRMS** (m/z): $[M+H]^+$ calcd. for $C_{18}H_{15}NH^+$, 246.1277; found, 246.1284.

1-Vinyl-1*H*-pyrrole (2d)

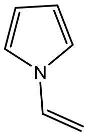

Yield, %: 79 (by NMR); 73 (isolated). **^{1}H NMR** (400 MHz, DMSO-d_6) δ 7.10 (t, *J* = 4.0 Hz, 2H), 7.02 (dd, *J* = 16.0, 8.0 Hz, 1H), 6.14 (t, *J* = 4.0 Hz, 1H), 5.23 (d, *J* = 16.0 Hz, 1H), 4.62 (d,

J = 8.0 Hz, 1H); ^{13}**C NMR** (101 MHz, DMSO-d_6) δ 133.2, 118.8, 109.8, 96.6; **HRMS** (m/z): $[M+H]^+$ calcd. for $C_6H_7NH^+$, 94.0651; found, 94.0655.

3,5-Dimethyl-1-vinyl-1*H*-pyrazole (2e)

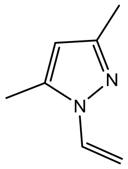

Yield, %: 72 (by NMR); 67 (isolated). ^{1}**H NMR** (400 MHz, DMSO-d_6) δ 7.07 (dd, *J* = 16.0, 8.0 Hz, 1H), 5.93 (s, 1H), 5.43 (d, *J* = 16.0 Hz, 1H), 4.70 (d, *J* = 8.0 Hz, 1H), 2.25 (s, 3H), 2.13 (s, 3H); ^{13}**C NMR** (101 MHz, DMSO-d_6) δ 148.6, 139.2, 129.9, 106.5, 98.5, 13.4, 10.3; **HRMS** (m/z): $[M+H]^+$ calcd. for $C_7H_{10}N_2H^+$, 123.0917; found, 123.0919.

1-Vinyl-1*H*-indole (2f)

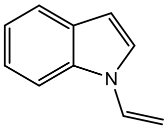

Yield ,%: 89 (by NMR); 79 (isolated). ^{1}**H NMR** (400 MHz, DMSO-d_6) δ 7.82 (s, 1H), 7.69 (d, *J* = 8.0 Hz, 1H), 7.59 (d, *J* = 8.0 Hz, 1H), 7.50 (dd, *J* = 16.0, 8.0 Hz, 1H), 7.22 (t, *J* = 8.0 Hz, 1H), 7.11 (t, *J* = 8.0 Hz, 1H), 6.66 (s, 1H), 5.39 (d, *J* = 12.0 Hz, 1H), 4.76 (d, *J* = 8.0 Hz, 1H); ^{13}**C NMR** (101 MHz, DMSO-d_6) δ 135.1, 129.9, 128.6, 124.0, 122.4, 120.7, 120.5, 110.0, 104.7, 96.4; **HRMS** (m/z): $[M+H]^+$ calcd. for $C_{10}H_9NH^+$, 144.0808; found, 144.0812.

2-Methyl-1-vinyl-*1H*-indole (2g)

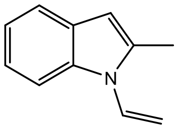

Yield, %: 78 (by NMR); 67 (isolated). ^{1}**H NMR** (400 MHz, DMSO-d_6) δ 7.64 (d, *J* = 8.0 Hz, 1H), 7.47 (d, *J* = 8.0 Hz, 1H), 7.21 (dd, *J* = 16.0, 8.0 Hz, 1H), 7.15−7.12 (m, 1H), 7.08−7.05 (m, 1H), 6.34 (s, 1H), 5.41 (d, *J* = 16.0 Hz, 1H), 5.05 (d, *J* = 8.0 Hz, 1H), 2.45 (s, 3H); ^{13}**C NMR** (101 MHz, DMSO-d_6) δ 136.4, 135.5, 130.3, 128.5, 121.5, 120.4, 119.5, 110.9, 102.6, 13.4; **HRMS** (m/z): $[M+H]^+$ calcd. for $C_{11}H_{11}NH^+$, 158.0964; found, 158.0970.

2,3-Dimethyl-1-vinyl-1*H*-indole (2h)

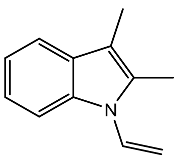

Yield, %: 86 (by NMR); 74 (isolated). ^{1}**H NMR** (400 MHz, DMSO-d_6) δ 7.62 (d, *J* = 8.0 Hz, 1H), 7.45 (d, *J* = 8.0 Hz, 1H), 7.20 (dd, *J* = 16.0, 8.0 Hz, 1H), 7.16−7.12 (m, 1H), 7.10−7.06 (m, 1H), 5.34 (d, *J* = 16.0 Hz, 1H), 4.97 (d, *J* = 8.0 Hz, 1H), 2.37 (s, 3H), 2.18 (s, 3H); ^{13}**C NMR**

(101 MHz, DMSO-d_6) δ 134.6, 132.0, 130.5, 129.4, 121.6, 120.0, 117.9, 110.7, 108.4, 101.3, 10.8, 8.4; **HRMS** (m/z): $[M+H]^+$ calcd. for $C_{12}H_{13}NH^+$, 172.1121; found, 172.1126.

7-Ethyl-1-vinyl-1*H*-indole (2i)

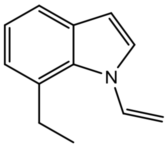

Yield, %: 85 (by NMR); 77 (isolated). **^{1}H NMR** (400 MHz, acetone-d_6) δ 7.67 (dd, *J* = 12.0, 8.0 Hz, 1H), 7.56 (d, *J* = 4.0 Hz, 1H), 7.44−7.40 (m, 1H), 7.02−7.00 (m, 2H), 6.61 (d, *J* = 4.0 Hz, 1H), 5.27 (d, *J* = 16.0 Hz, 1H), 4.82 (d, *J* = 8.0, Hz, 1H), 3.07 (q, *J* = 8.0 Hz, 2H), 1.32 (t, *J* = 8.0 Hz, 3H); **^{13}C NMR** (101 MHz, acetone-d_6) δ 134.5, 134.2, 131.4, 128.7, 126.1, 124.9, 121.5, 120.0, 105.8, 99.8, 27.0, 16.1.

2-Phenyl-1-vinyl-1*H*-indole (2j)

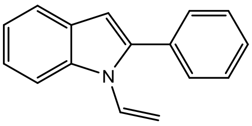

Yield, %: 37 (by NMR); 16 (isolated). **^{1}H NMR** (400 MHz, DMSO-d_6) δ 7.76 (d, *J* = 8.0 Hz, 1H), 7.62 (d, *J* = 8.0 Hz, 1H), 7.57−7.55 (m, 2H), 7.53−7.49 (m, 2H), 7.46−7.42 (m, 1H), 7.27−7.23 (m, 1H), 7.18−7.14 (m, 1H), 7.07 (dd, *J* = 16.0, 8.0 Hz, 1H), 6.72 (s, 1H), 5.28 (d, *J* = 16.0 Hz, 1H), 5.13 (d, *J* = 8.0 Hz, 1H); **^{13}C NMR** (101 MHz, DMSO-d_6) δ 139.8, 136.4, 131.9, 131.1, 129.1, 128.6, 128.4, 128.2, 122.7, 121.0, 120.5, 111.5, 105.4, 104.4; **HRMS** (m/z): $[M+H]^+$ calcd. for $C_{16}H_{13}NH^+$, 220.1121; found, 220.1120.

6-Chloro-1-vinyl-1*H*-indole (2k)

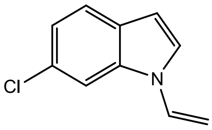

Yield, %: 55 (by NMR); 39 (isolated). **^{1}H NMR** (400 MHz, DMSO-d_6) δ 7.88−7.87 (m, 2H), (d, *J* = 4.0 Hz, 2H), 7.59 (d, *J* = 12.0 Hz, 1H), 7.54 (dd, *J* = 16.0, 12.0 Hz, 1H), 7.12 (dd, *J* = 12.0, 4.0 Hz, 1H), 6.69 (d, *J* = 4.0 Hz, 1H), 5.42 (d, *J* = 16.0 Hz, 1H), 4.79 (d, *J* = 8.0 Hz, 1H); **^{13}C NMR** (101 MHz, DMSO-d_6) δ 135.5, 129.7, 127.4, 127.2, 124.9, 122.0, 120.8, 110.1, 104.8, 97.4; **HRMS** (m/z): $[M+H]^+$ calcd. for $C_{10}H_8NClH^+$, 178.0418; found, 178.0409.

9-Vinyl-2,3,4,9-tetrahydro-1*H*-carbazole (2l)

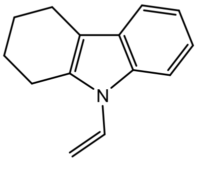

Yield, %: 84 (by NMR); 79 (isolated). **^{1}H NMR** (400 MHz, DMSO-d_6) δ 7.63 (d, *J* = 8.0 Hz, 1H), 7.41 (d, *J* = 8.0 Hz, 1H), 7.23−7.08 (m, 3H), 5.26 (d, *J* = 16.0 Hz, 1H), 4.87 (d, *J* = 8.0 Hz, 1H), 2.77−2.62 (m, 4H), 1.84−1.78 (m, 4H); **^{13}C NMR** (101 MHz, DMSO-d_6) δ 135.0, 134.8, 129.9, 128.0, 121.7, 120.1, 117.6, 111.6, 110.7, 99.6, 22.8, 22.2, 20.5; **HRMS** (m/z): $[M+H]^+$ calcd. for $C_{14}H_{15}NH^+$, 198.1277; found, 198.1277

9,9'-Divinyl-9*H*,9'*H*-3,3'-bicarbazole (2m)

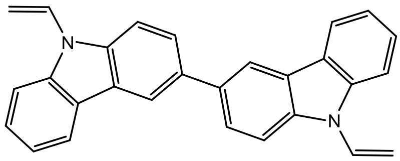

Yield, %: 32 (isolated). **^{1}H NMR** (400 MHz, DMSO-d_6) δ 8.67 (s, 2H), 8.35 (d, *J* = 8.0 Hz, 2H), 7.96 (br s, 4H), 7.88 (d, *J* = 8.0 Hz, 2H), 7.65 (dd, *J* = 16.0, 8.0 Hz, 2H), 7.53 (t, *J* = 8.0, 8.0 Hz, 2H), 7.35 (t, *J* = 8.0, 8.0 Hz, 2H), 5.65 (d, *J* = 16.0 Hz, 2H), 5.16 (d, *J* = 8.0 Hz, 2H); **^{13}C NMR** (101 MHz, DMSO-d_6): *δ* 139.2, 137.9, 133.6, 129.9, 126.7, 125.6, 124.1, 123.5, 120.9, 120.7, 118.4, 111.4, 111.0, 101.0; **HRMS** (m/z): $[M+H]^+$ calcd. for $C_{28}H_{20}N_2H^+$, 385.1699; found, 385.1689

2-Methyl-4-(4-methylpiperazin-1-yl)-10-vinyl-10*H*-benzo[*b*]thieno[2,3-*e*][1,4]diazepine (2n)

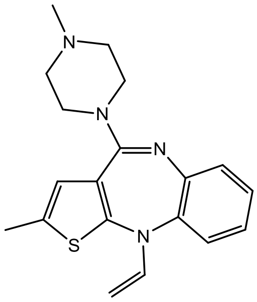

Yield, %: 44 (isolated). **^{1}H NMR** (400 MHz, DMSO-d_6) δ 7.11−7.07 (m, 1H), 7.04−7.02 (m, 1H), 6.98 (t, *J* = 8.0 Hz, 2H), 6.65 (dd, *J* = 16.0, 8.0 Hz, 1H), 6.56 (s, 1H), 4.51 (d, *J* = 16.0 Hz, 1H), 4.11 (d, *J* = 8.0 Hz, 1H), 3.46 (m, 4H), 2.45−2.42 (m, 2H), 2.39 (s, 3H), 2.33−2.30 (m, 2H), 2.21 (s, 3H); **^{13}C NMR** (101 MHz, DMSO-d_6): *δ* 155.3, 146.0, 143.6, 140.2, 138.9, 136.3, 127.1, 126.3, 124.6, 123.7, 123.2, 121.5, 87.4, 54.4, 46.0, 45.7, 15.6; **HRMS** (m/z): $[M+H]^+$ calcd. for $C_{19}H_{22}N_4SH^+$, 339.1638; found, 339.1640

NMR spectra

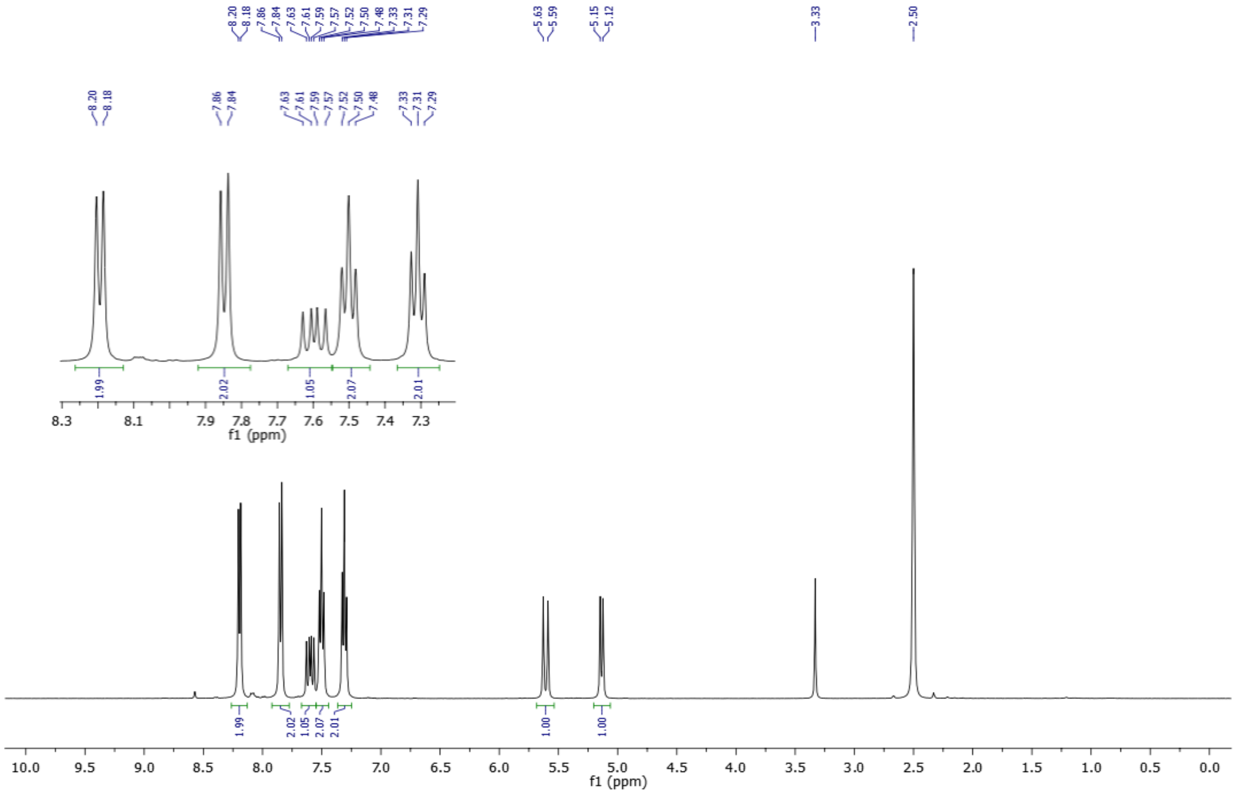

Figure S1. ^{1}H NMR spectrum of 9-vinyl-9*H*-carbazole **(2a)**

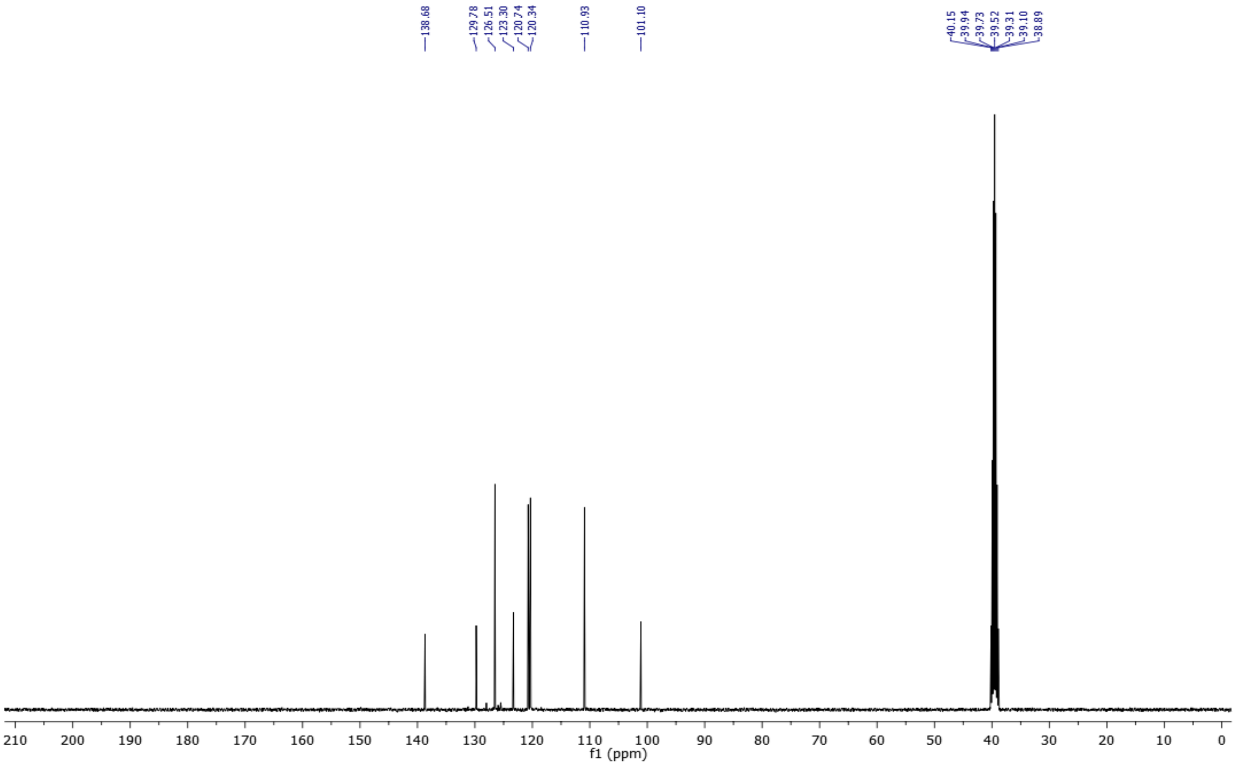

Figure S2. ^{13}C NMR spectrum of 9-vinyl-9*H*-carbazole **(2a)**

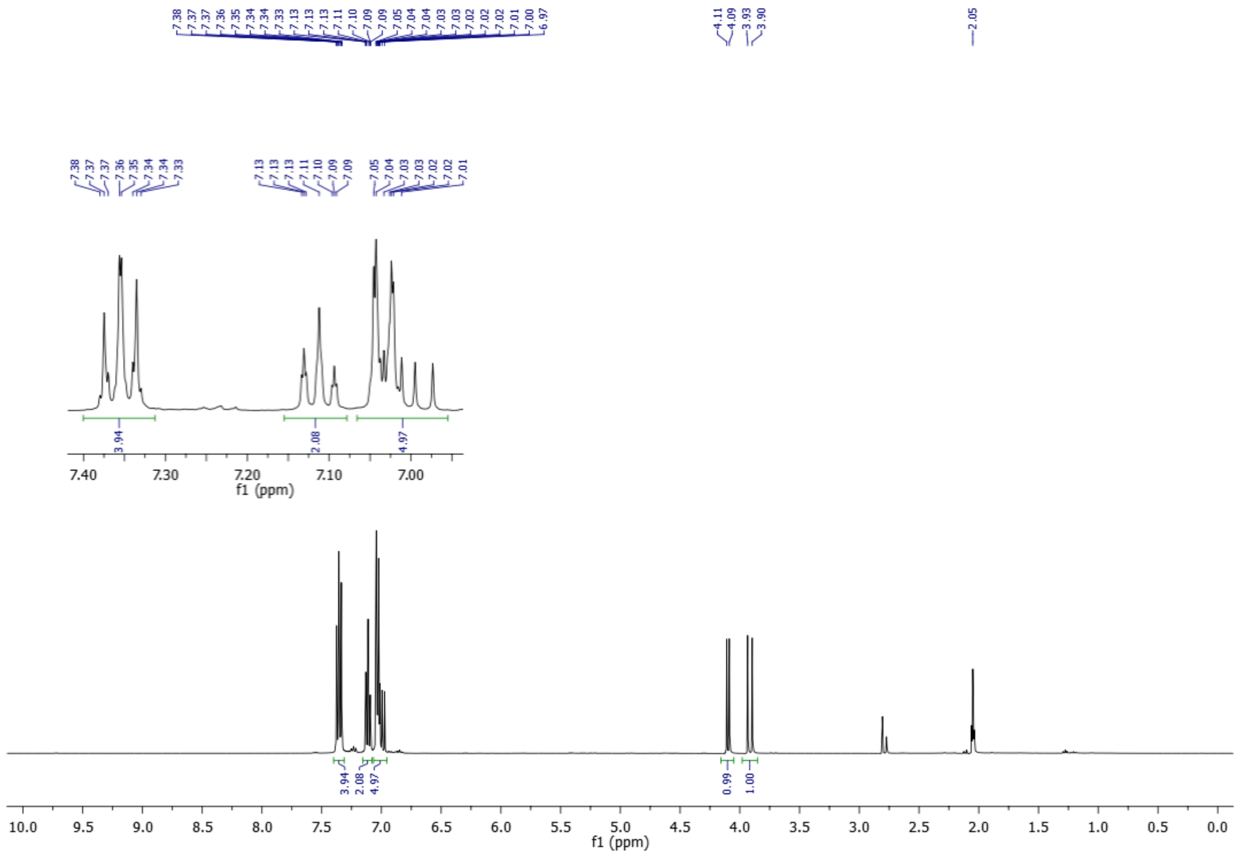

Figure S3. ^{1}H NMR spectrum of N,N-diphenylvinylamine **(2b)**

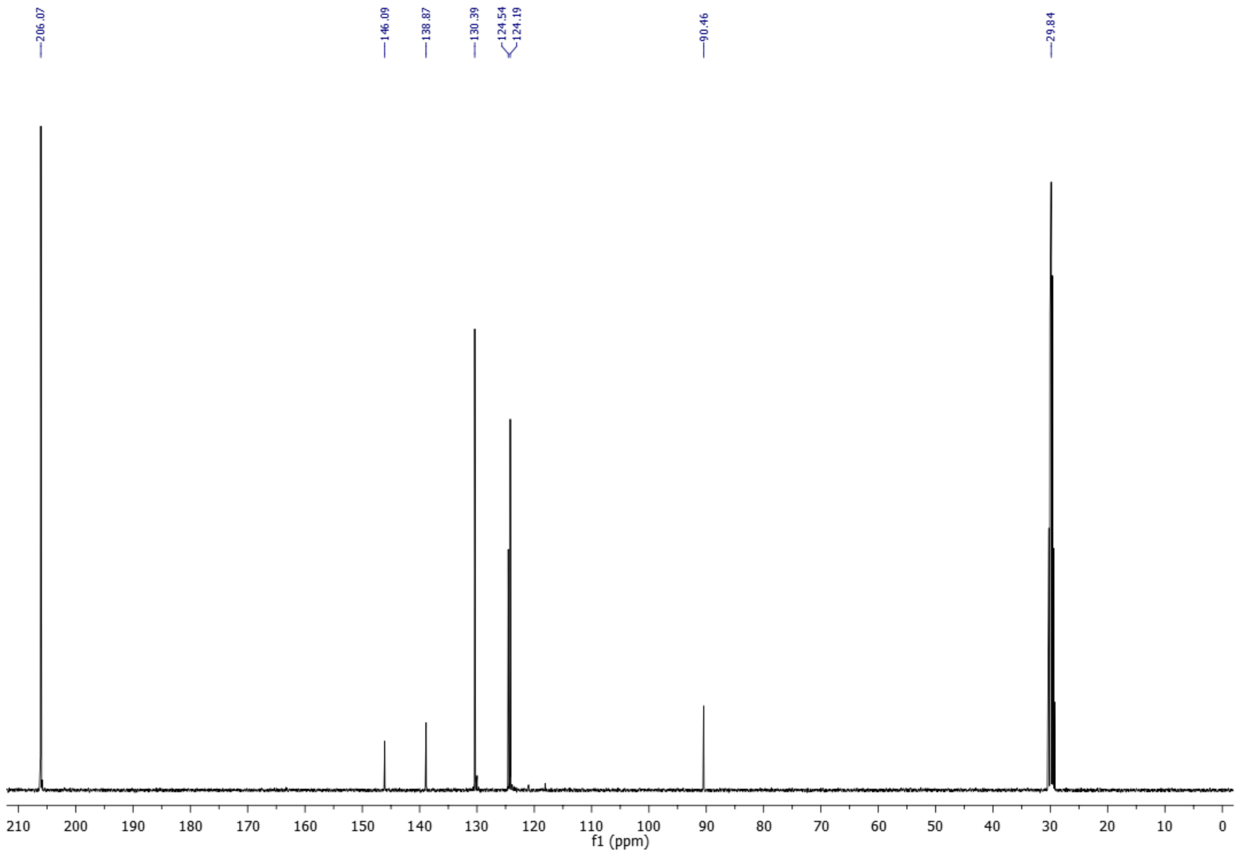

Figure S4. ^{13}C NMR spectrum of N,N-diphenylvinylamine **(2b)**

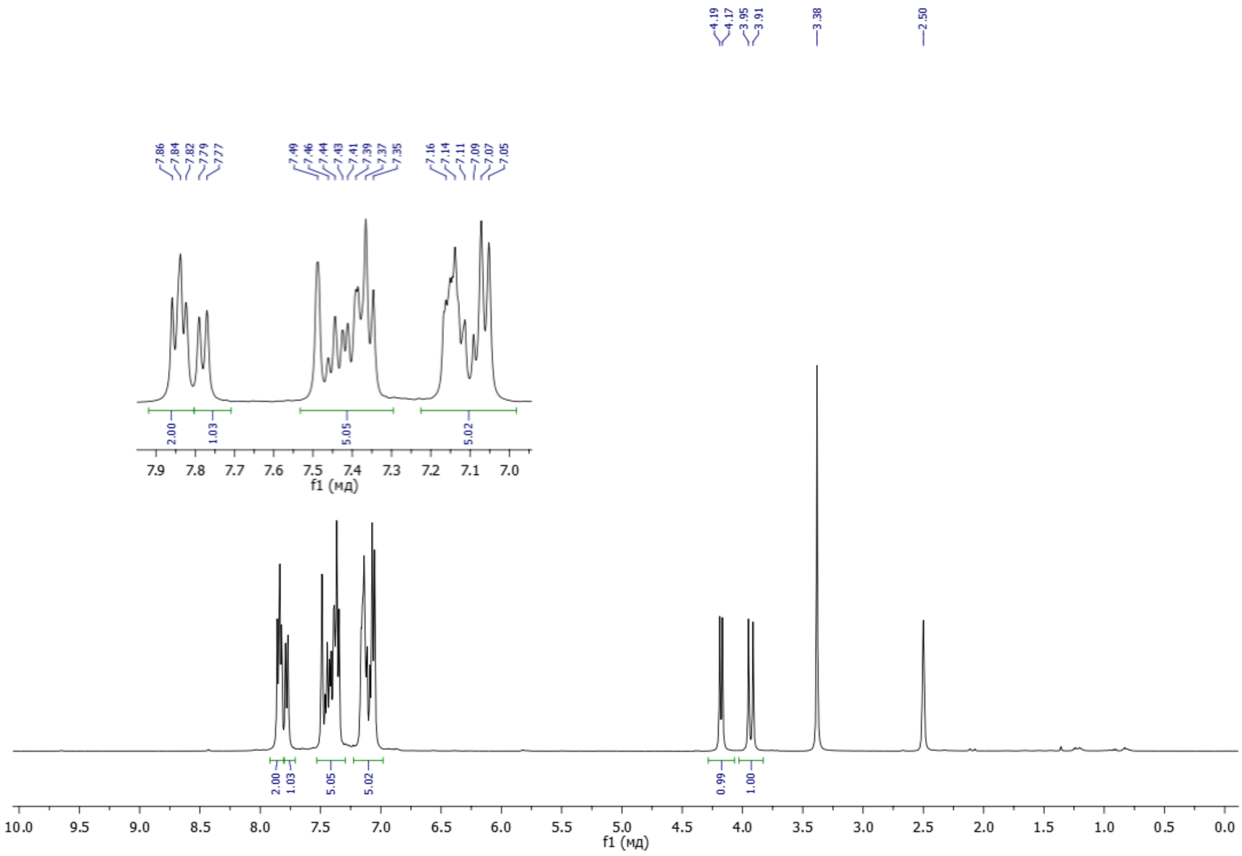

Figure S5. ^{1}H NMR spectrum of N-(β-naphthyl)-N-phenylvinylamine **(2c)**

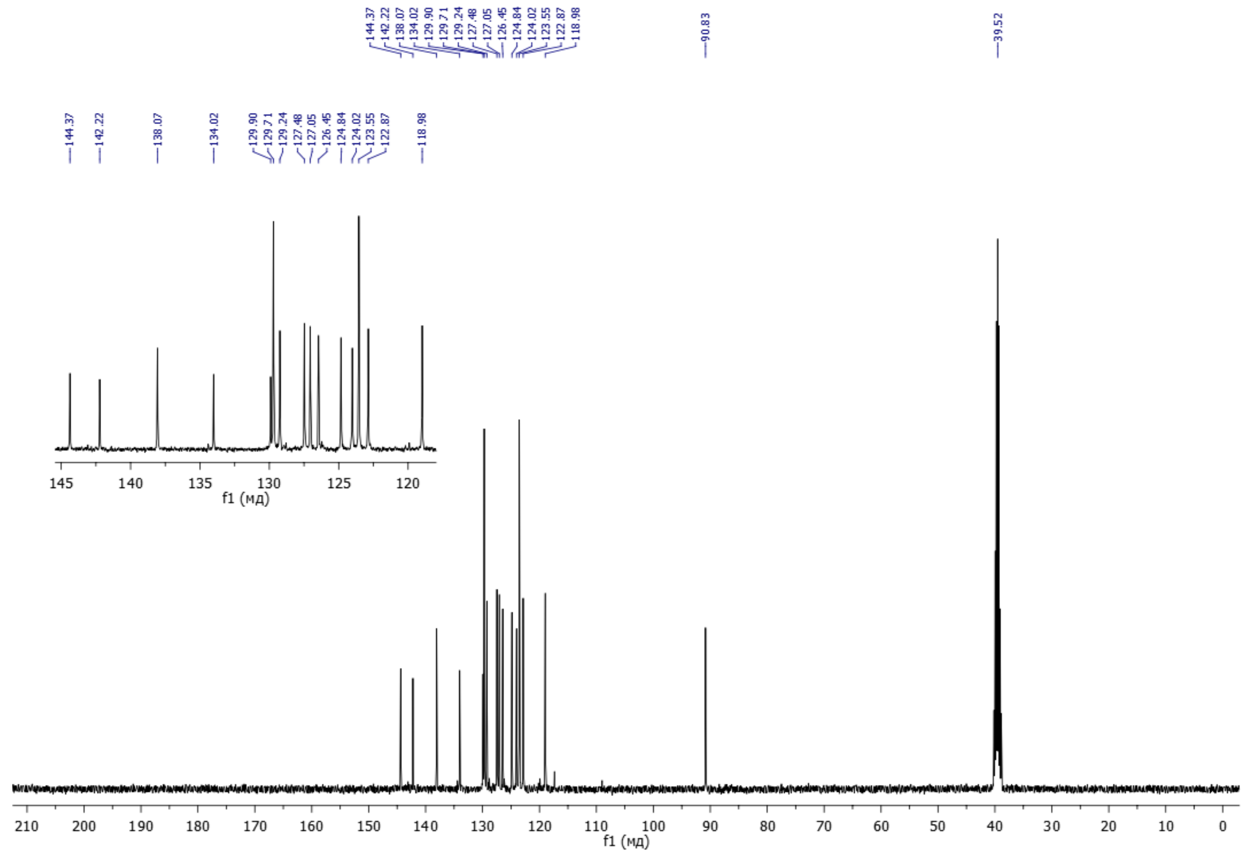

Figure S6. ^{13}C NMR spectrum of N-(β-naphthyl)-N-phenylvinylamine **(2c)**

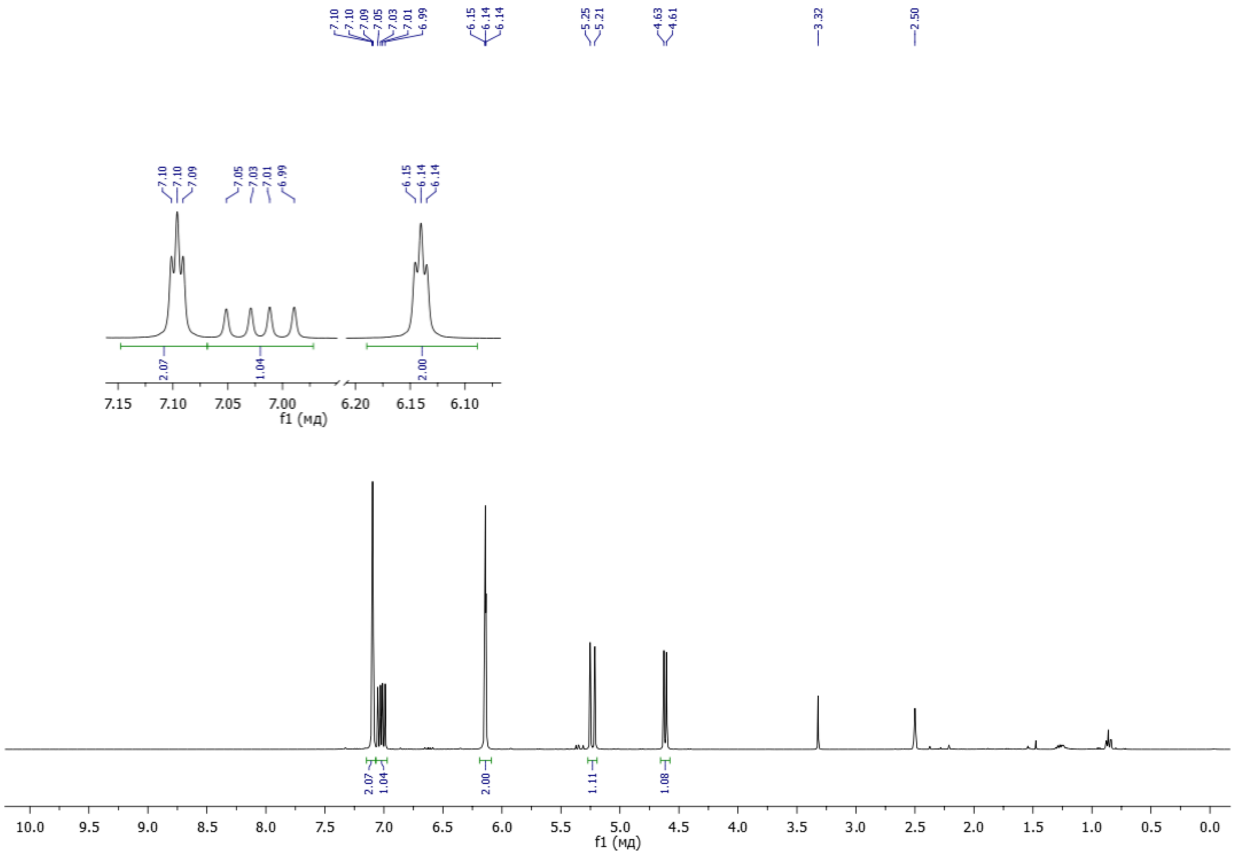

Figure S7. ^{1}H NMR spectrum of 1-vinyl-1*H*-pyrrole **(2d)**

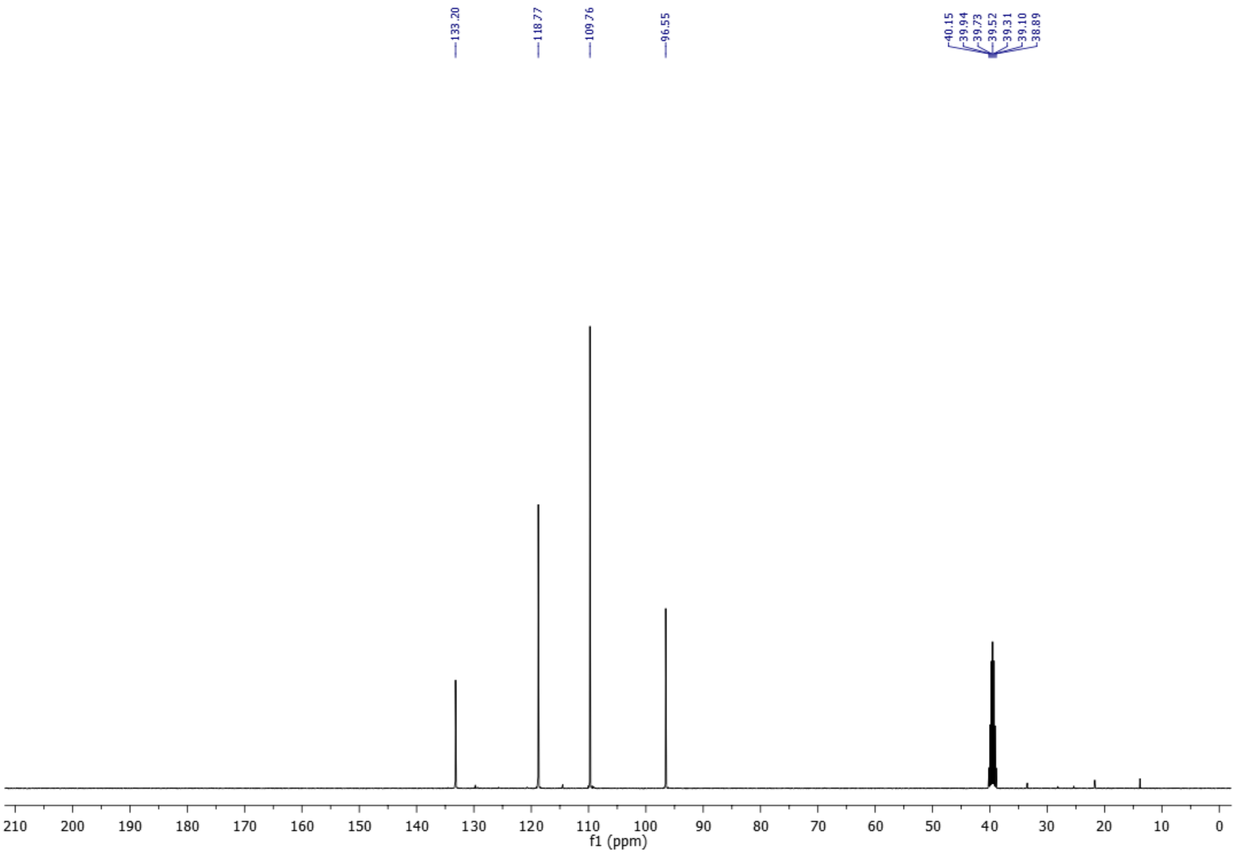

Figure S8. ^{13}C NMR spectrum of 1-vinyl-1*H*-pyrrole **(2d)**

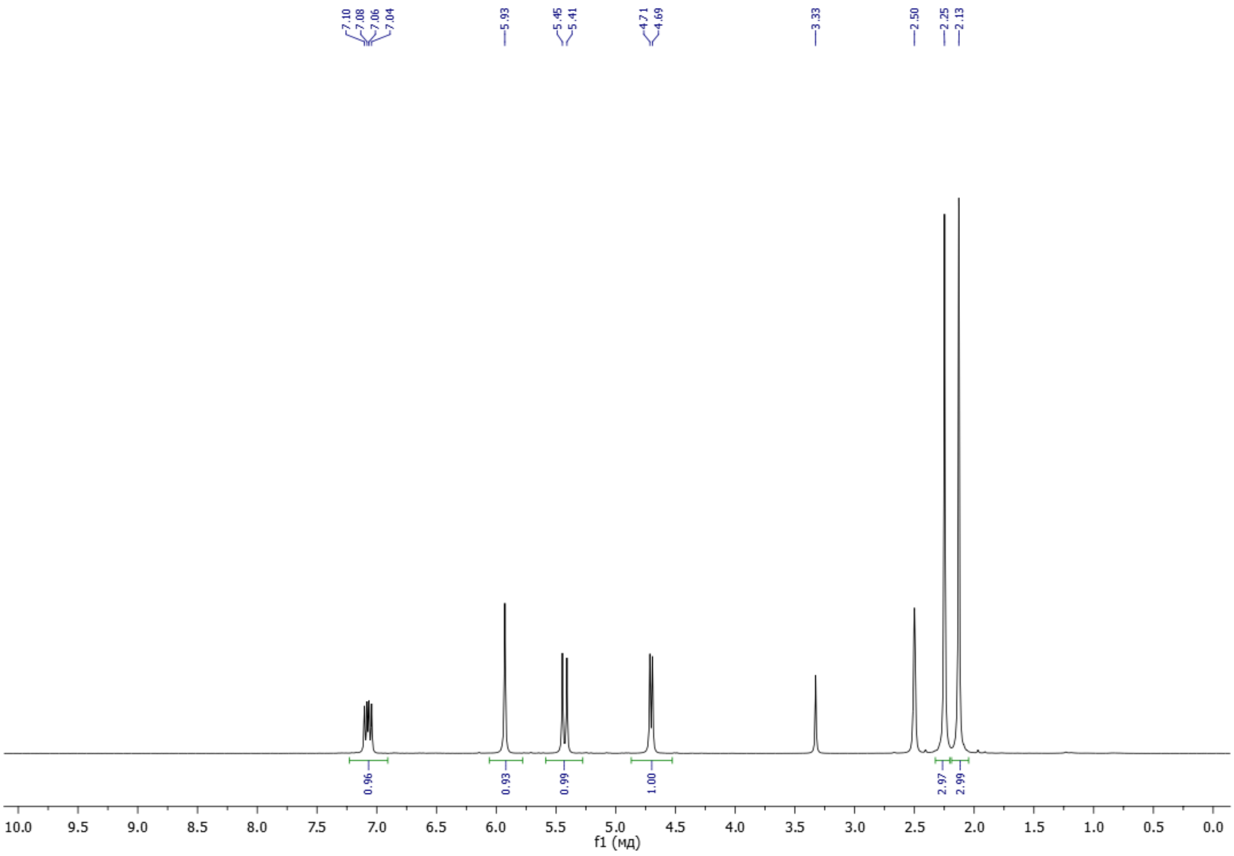

Figure S9. ^{1}H NMR spectrum of 3,5-dimethyl-1-vinyl-1*H*-pyrazole **(2e)**

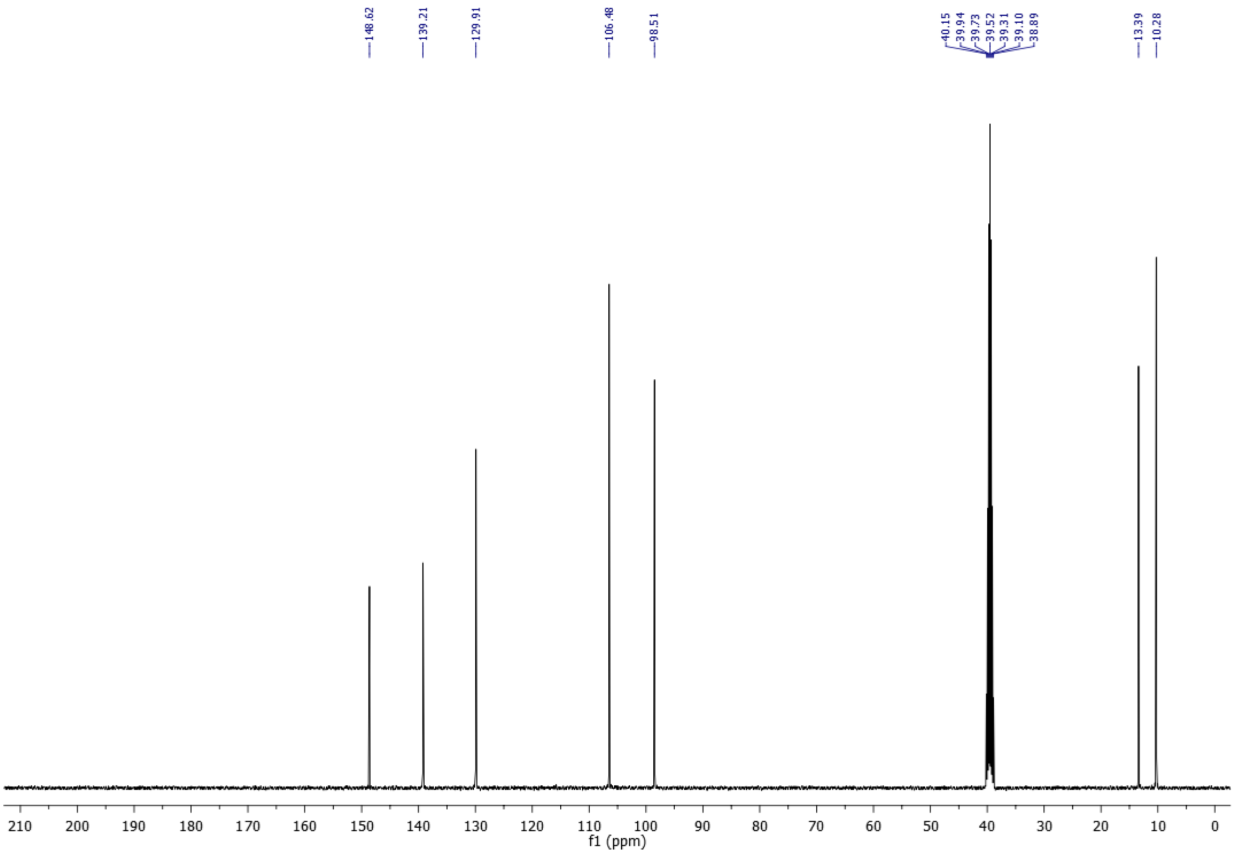

Figure S10. ^{13}C NMR spectrum of 3,5-dimethyl-1-vinyl-1*H*-pyrazole **(2e)**

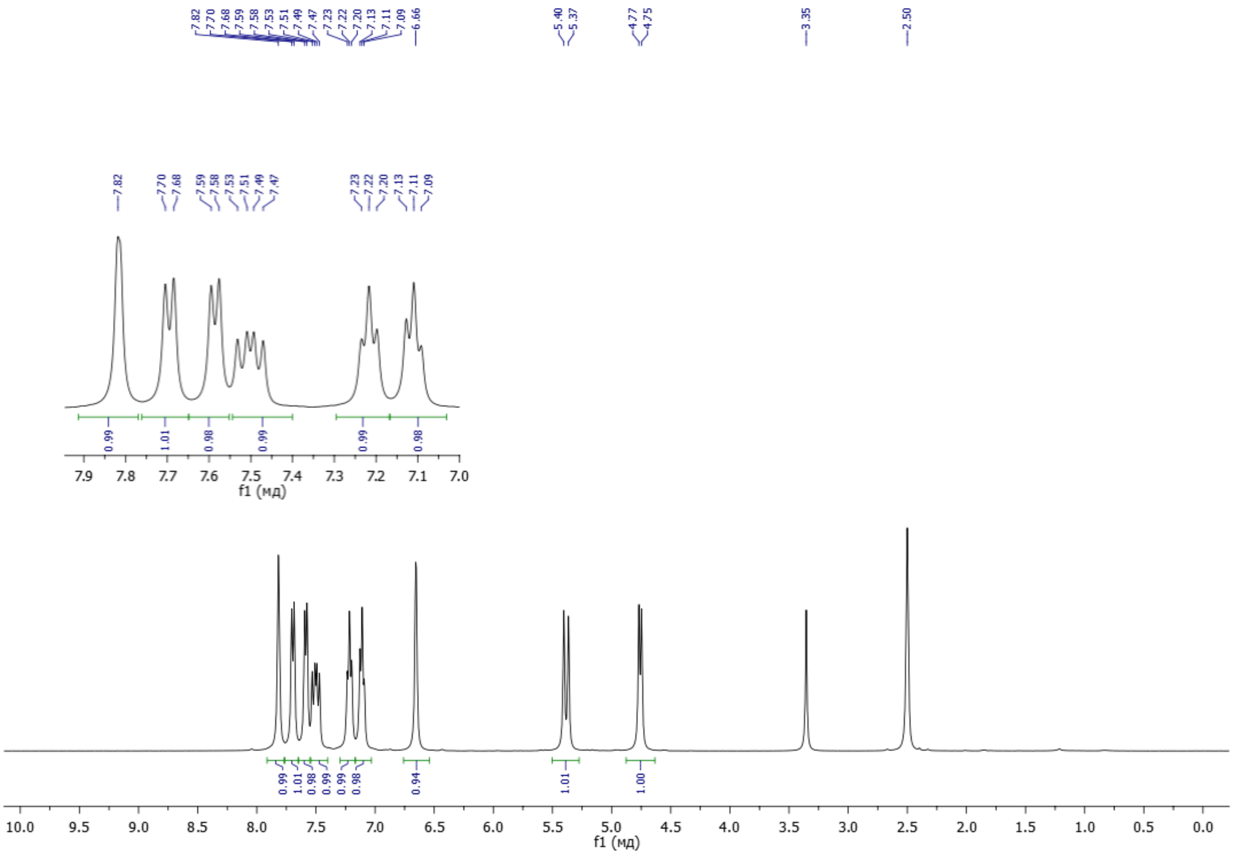

Figure S11. ^{1}H NMR spectrum of 1-vinyl-1*H*-indole **(2f)**

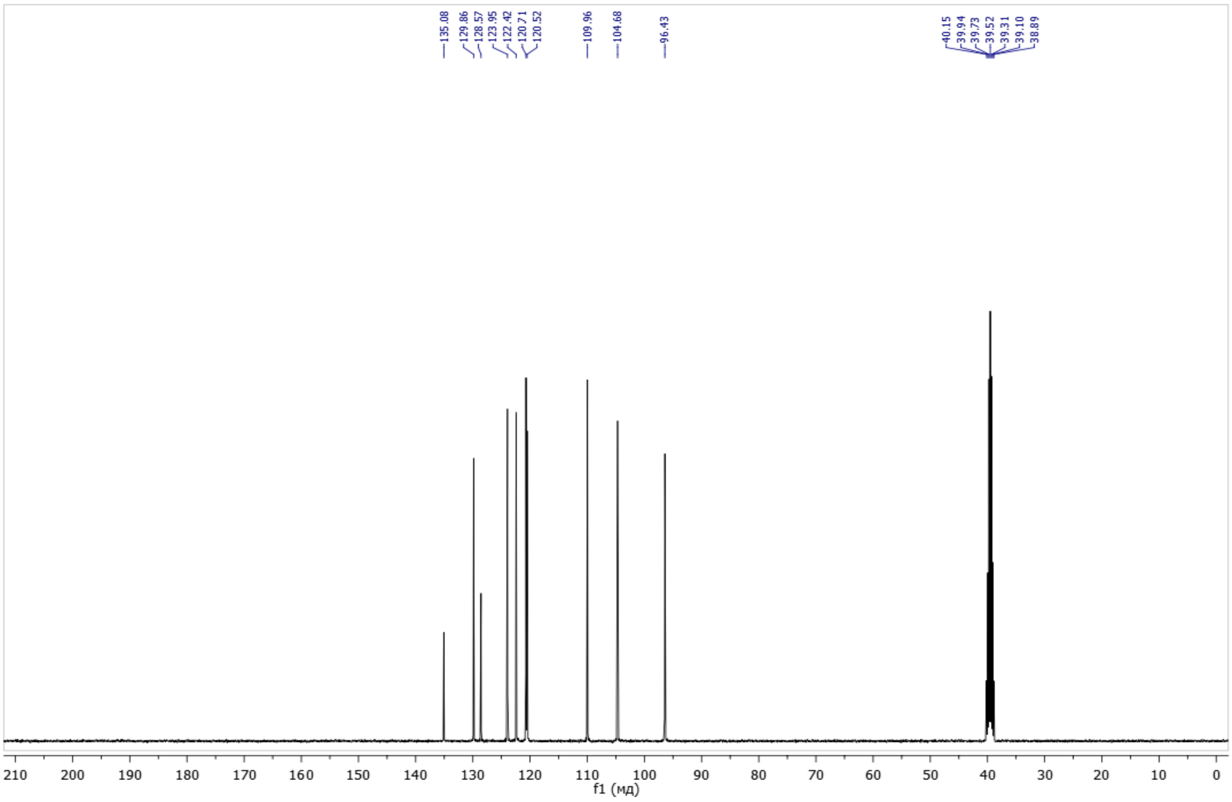

Figure S12. ^{13}C NMR spectrum of 1-vinyl-1*H*-indole **(2f)**

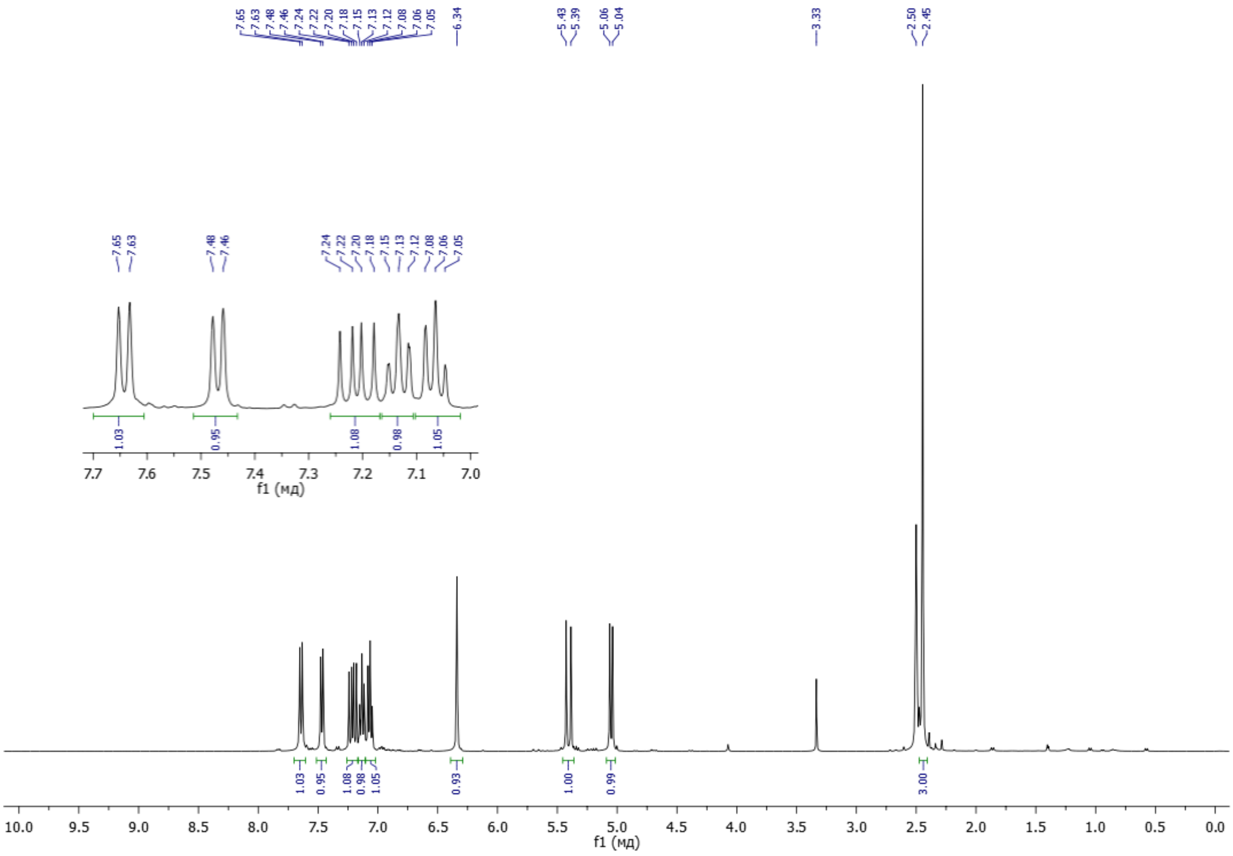

Figure S13. ^{1}H NMR spectrum of 2-methyl-1-vinyl-1*H*-indole **(2g)**

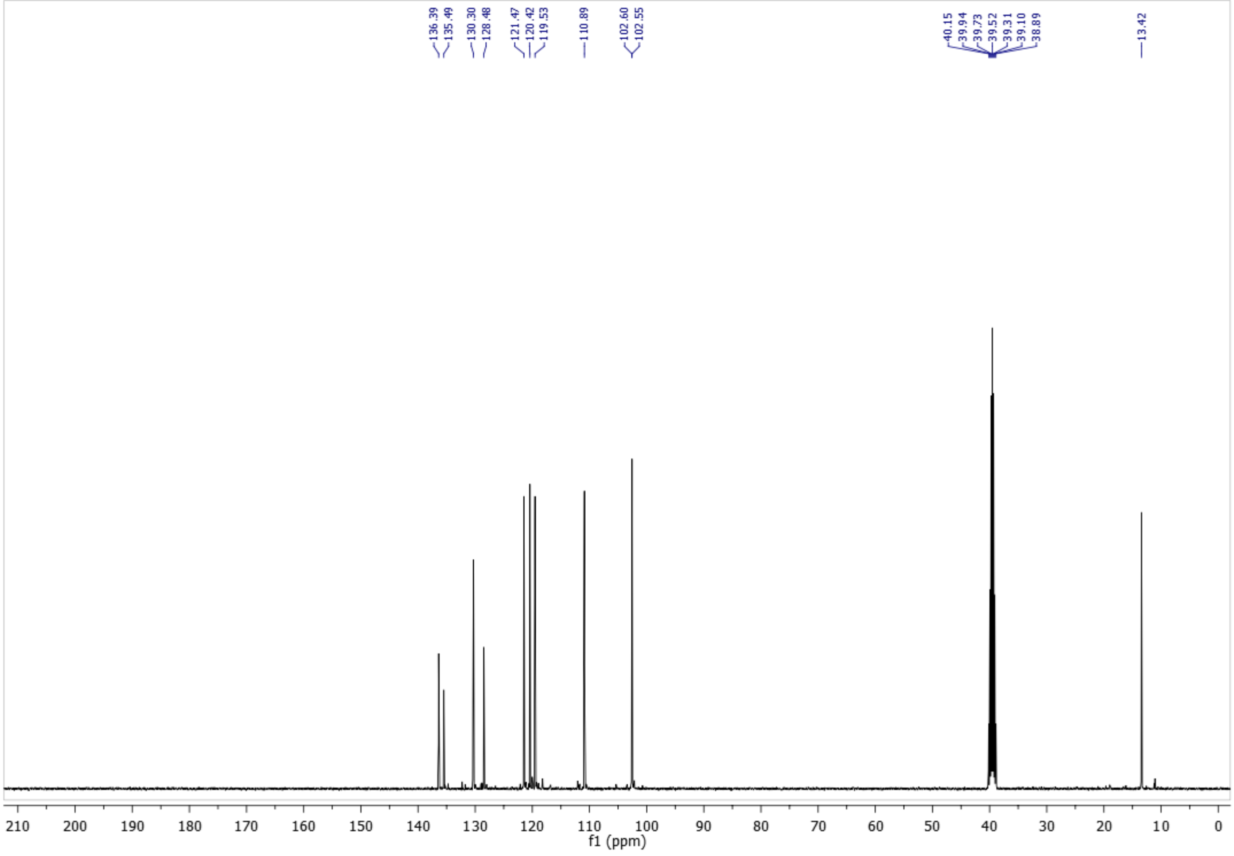

Figure S14. ^{13}C NMR spectrum of 2-methyl-1-vinyl-1*H*-indole **(2g)**

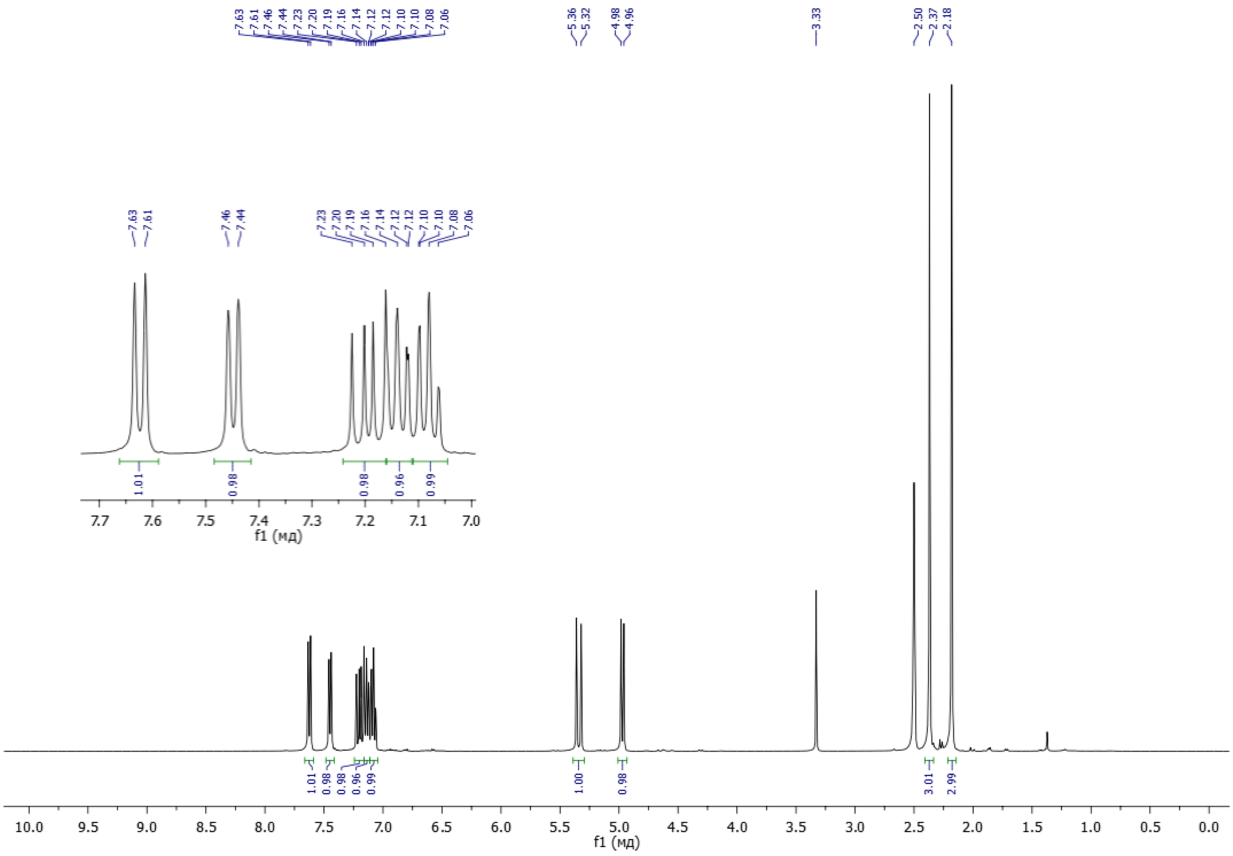

Figure S15. ^{1}H NMR spectrum of 2,3-dimethyl-1-vinyl-1*H*-indole **(2h)**

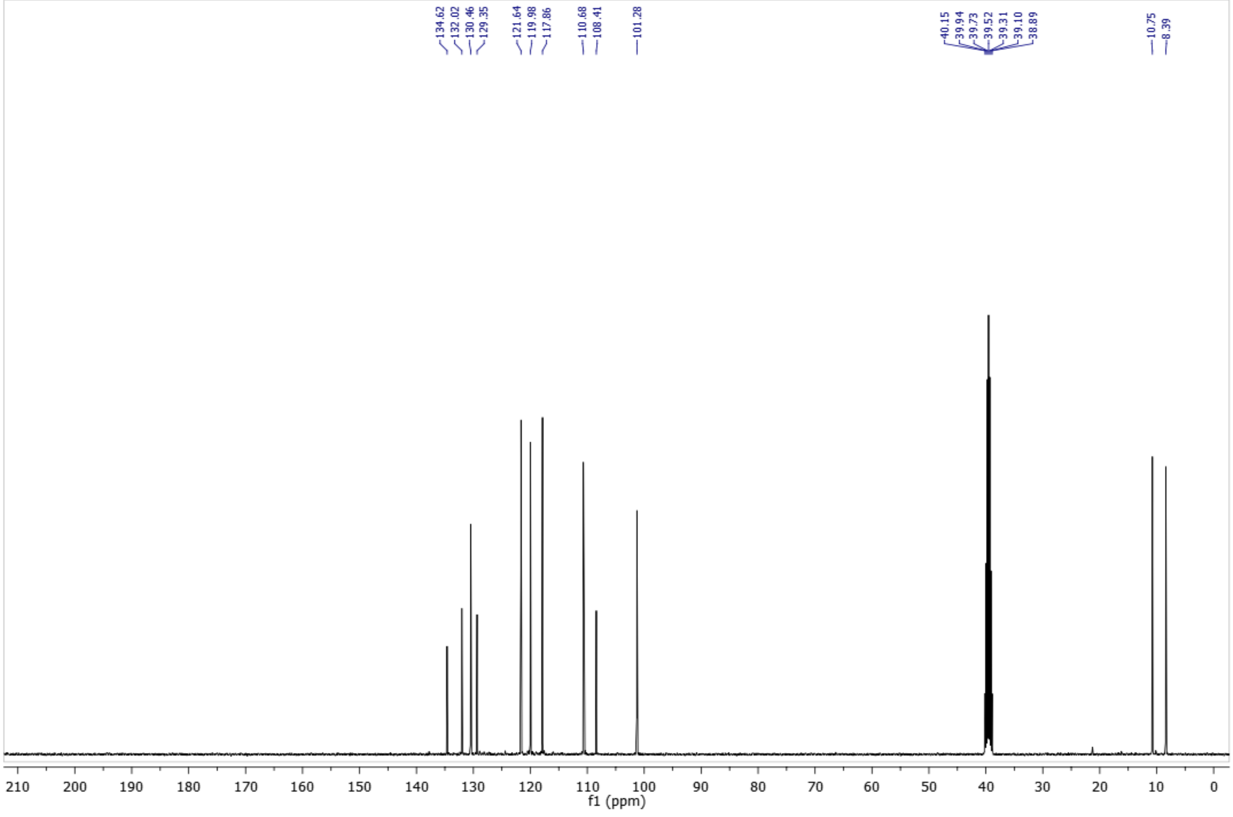

Figure S16. ^{13}C NMR spectrum of 2,3-dimethyl-1-vinyl-1*H*-indole **(2h)**

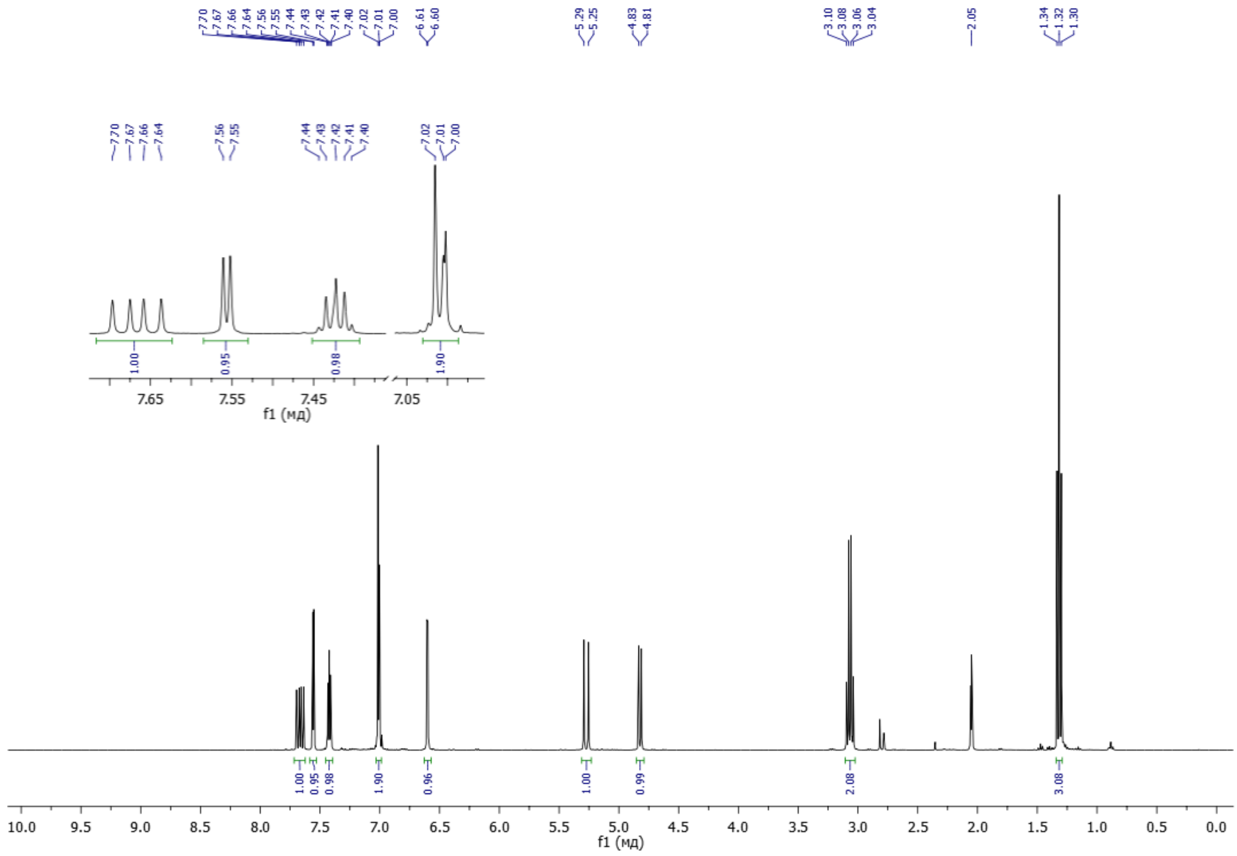

Figure S17. ^{1}H NMR spectrum of 7-ethyl-1-vinyl-1*H*-indole **(2i)**

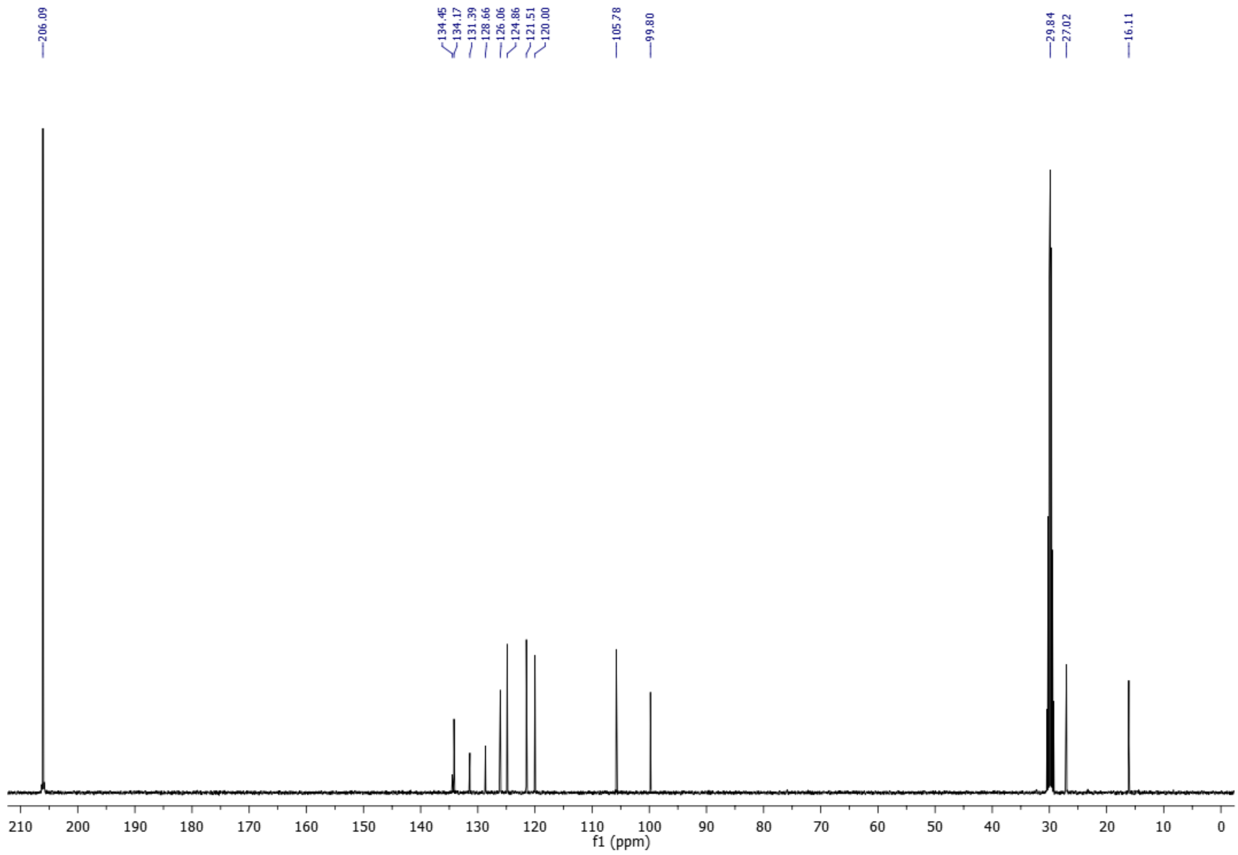

Figure S18. ^{13}C NMR spectrum of 7-ethyl-1-vinyl-1*H*-indole **(2i)**

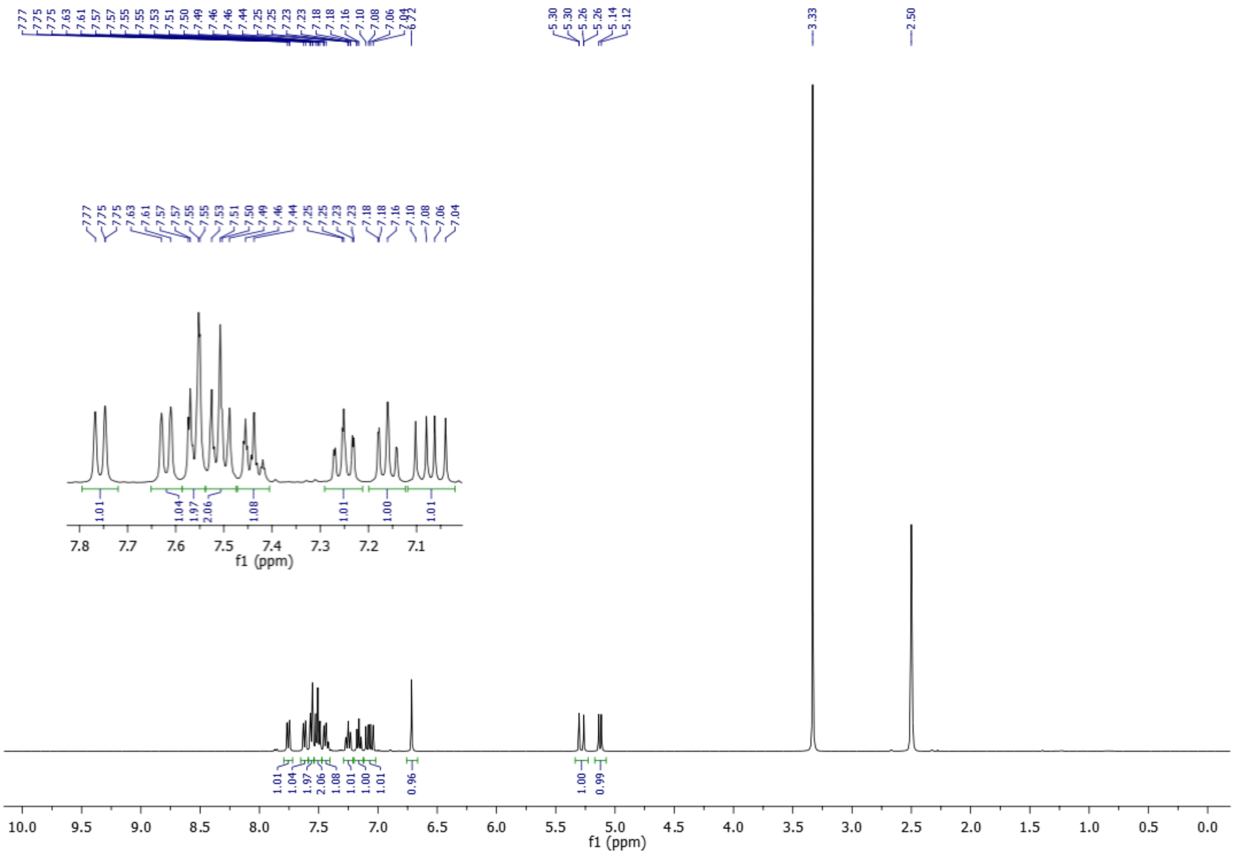

Figure S19. ^{1}H NMR spectrum of 2-phenyl-1-vinyl-1*H*-indole **(2j)**

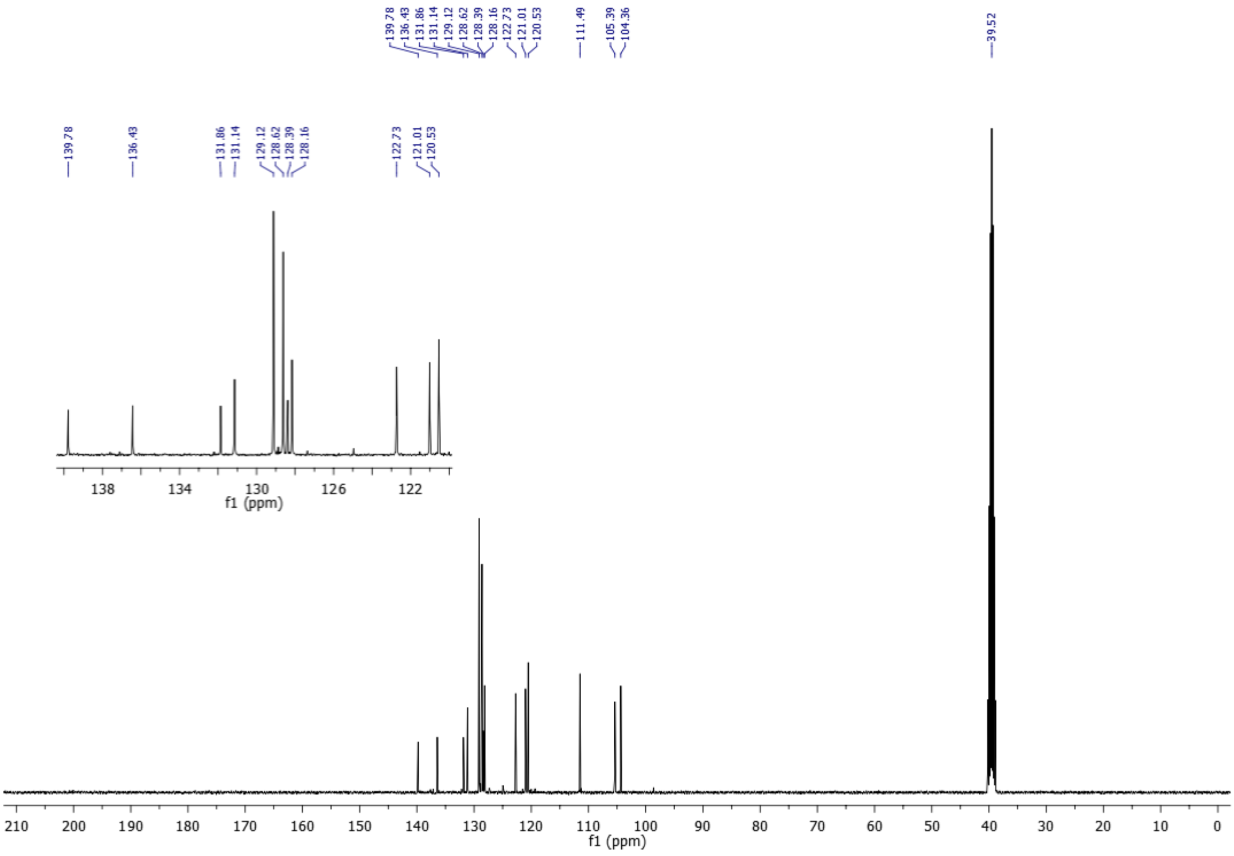

Figure S20. ^{13}C NMR spectrum of 2-phenyl-1-vinyl-1*H*-indole **(2j)**

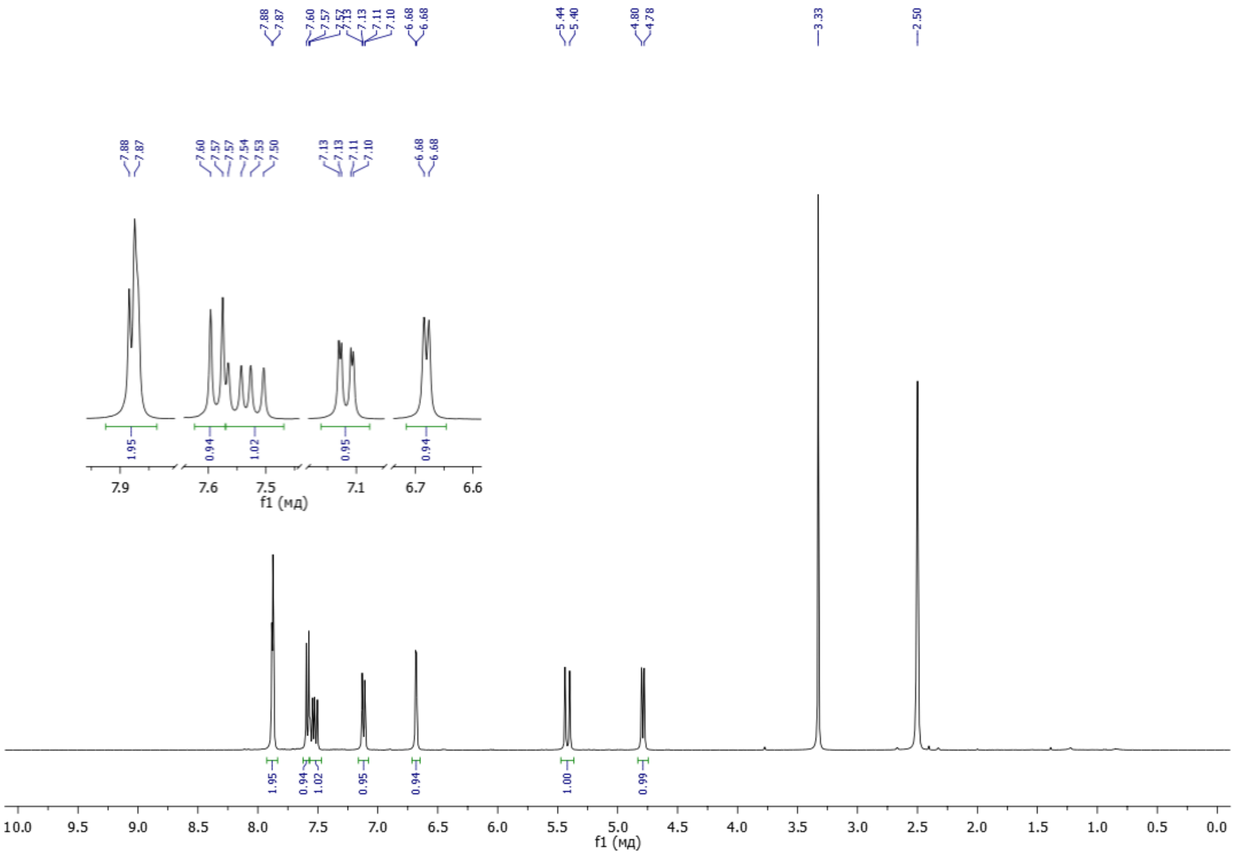

Figure S21. ^{1}H NMR spectrum of 6-chloro-1-vinyl-1*H*-indole **(2k)**

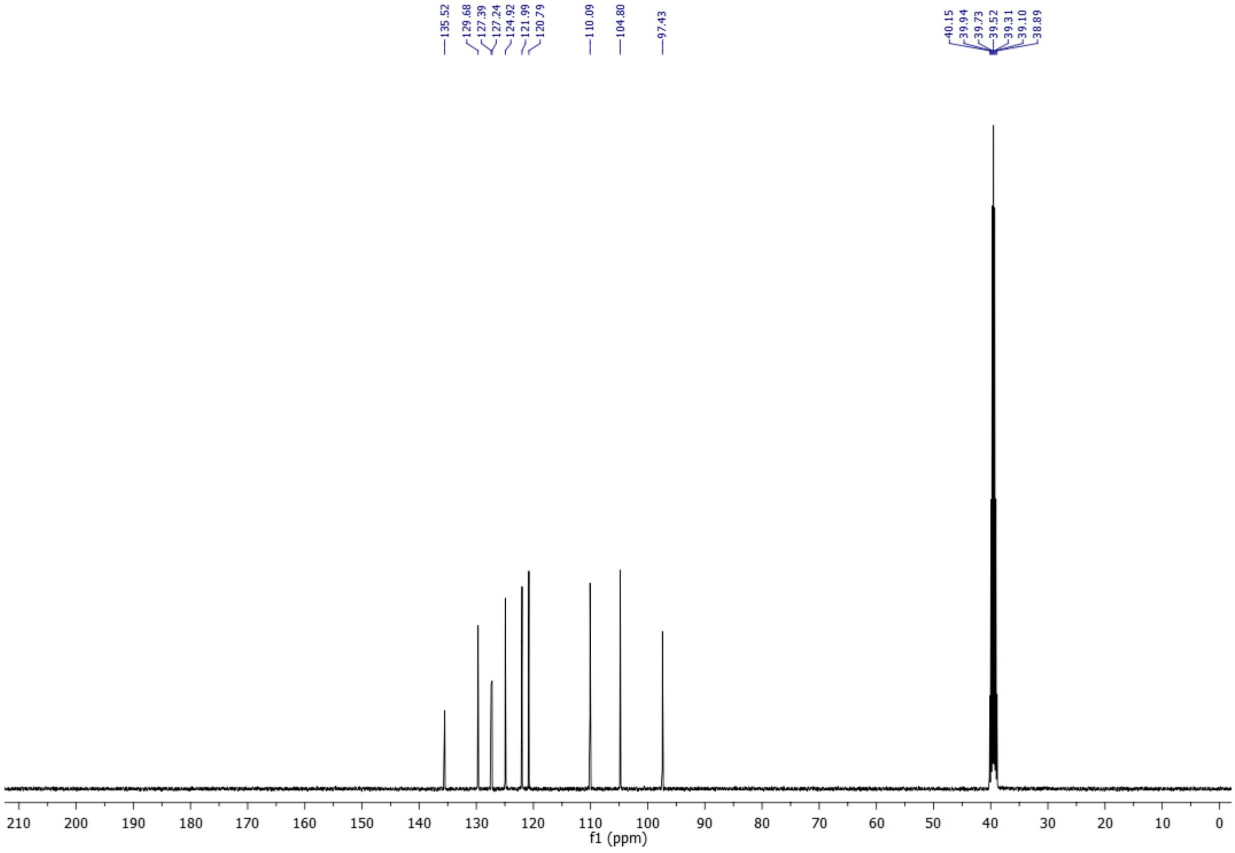

Figure S22. ^{13}C NMR spectrum of 6-chloro-1-vinyl-1*H*-indole **(2k)**

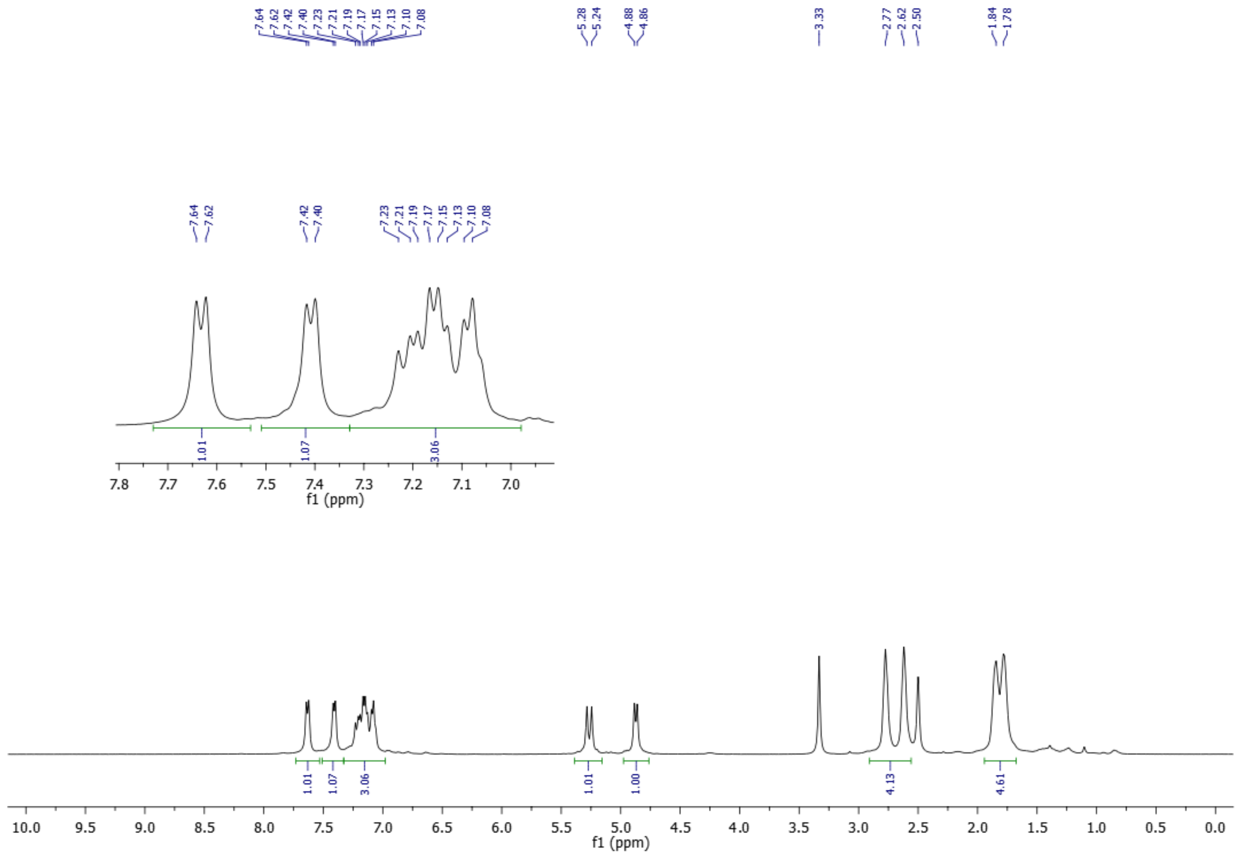

Figure S23. ^{1}H NMR spectrum of 9-vinyl-2,3,4,9-tetrahydro-1*H*-carbazole **(2l)**

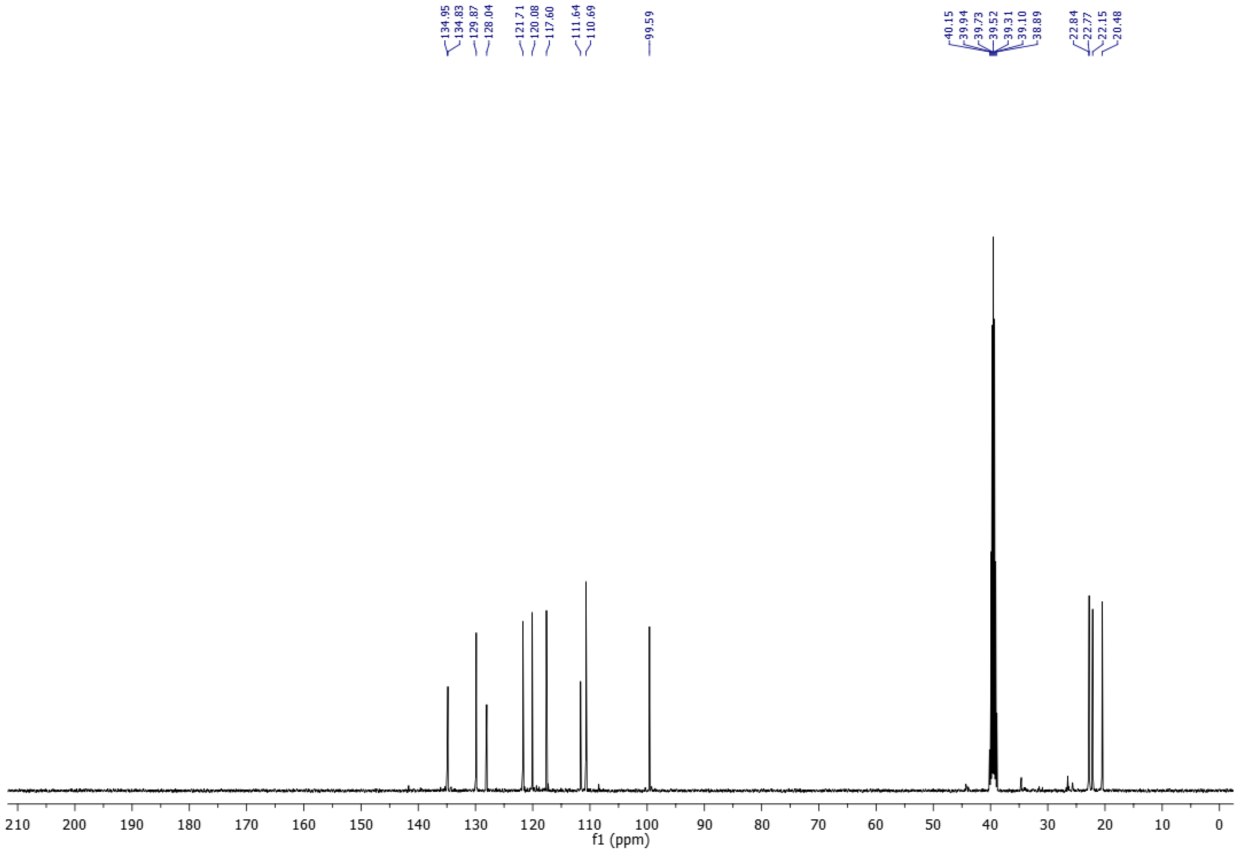

Figure S24. ^{13}C NMR spectrum of 9-vinyl-2,3,4,9-tetrahydro-1*H*-carbazole **(2l)**

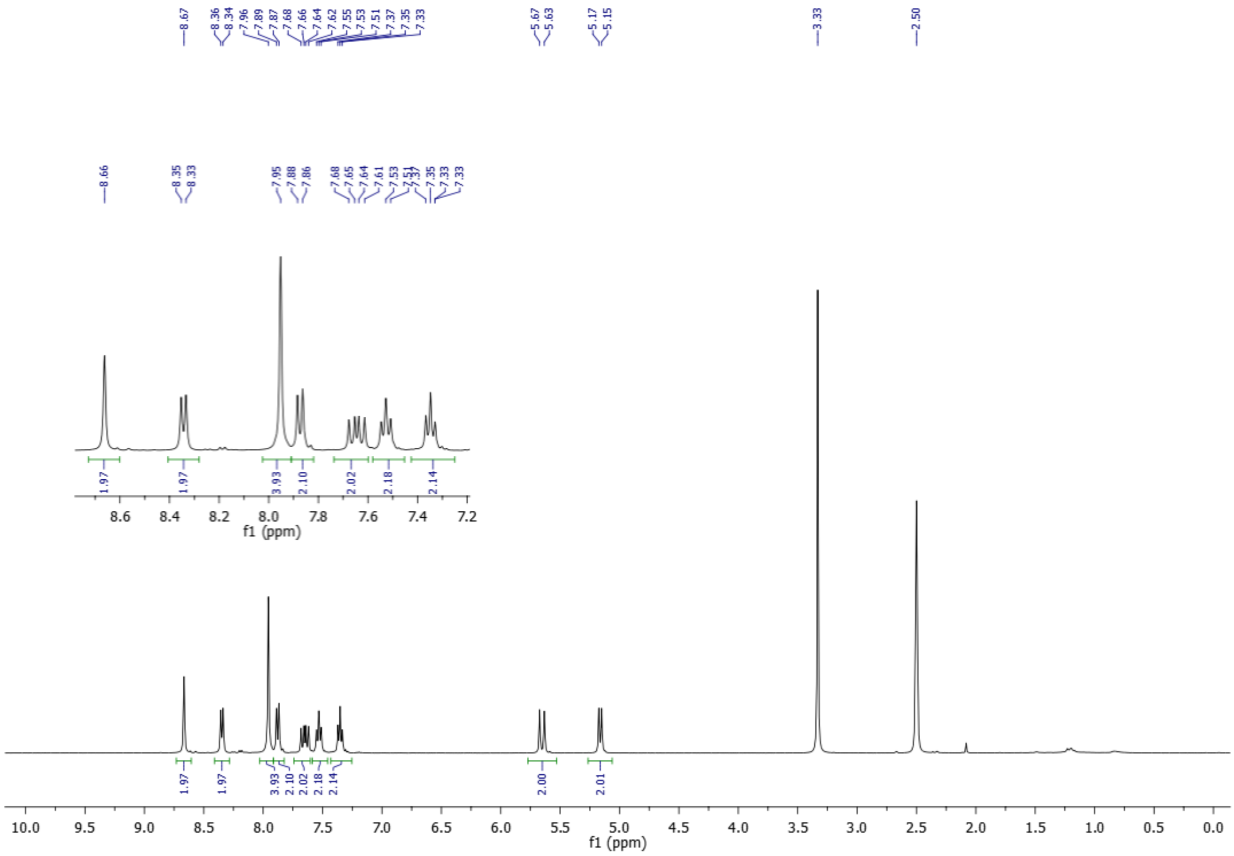

Figure S25. ^{1}H NMR spectrum of 9,9'-divinyl-9*H*,9'*H*-3,3'-bicarbazole **(2m)**

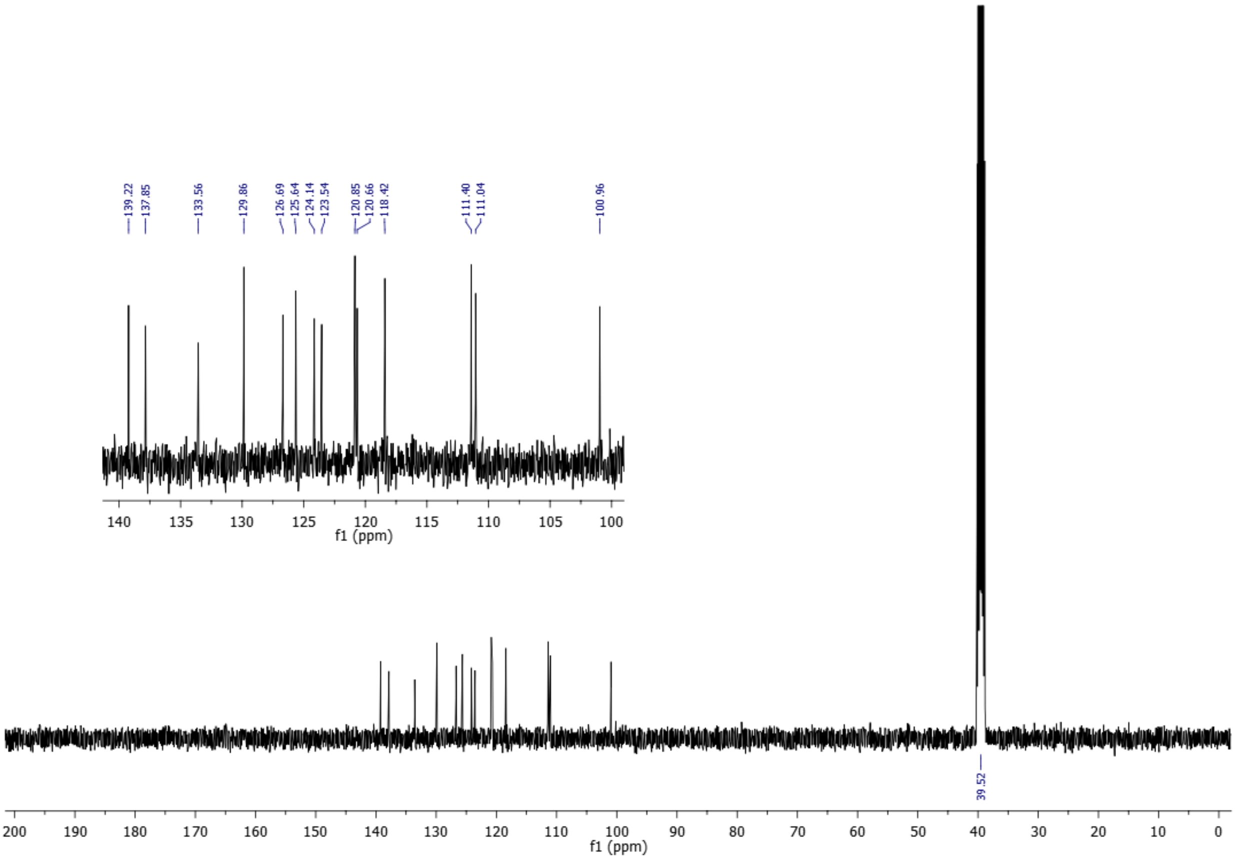

Figure S26. ^{13}C NMR spectrum of 9,9'-divinyl-9*H*,9'*H*-3,3'-bicarbazole **(2m)**

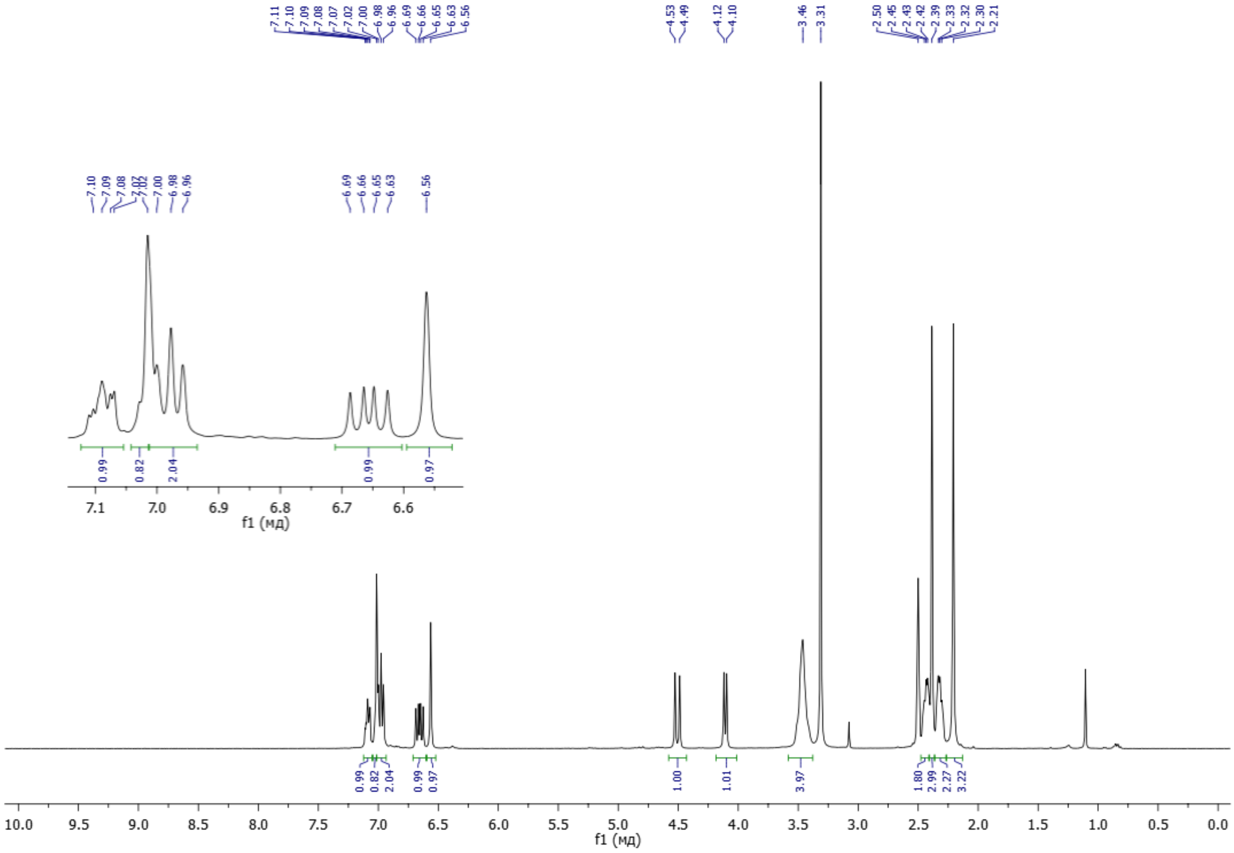

Figure S27. ^{1}H NMR spectrum of 2-methyl-4-(4-methylpiperazin-1-yl)-10-vinyl-10*H*-benzo[*b*]thieno[2,3-*e*][1,4]diazepine **(2n)**

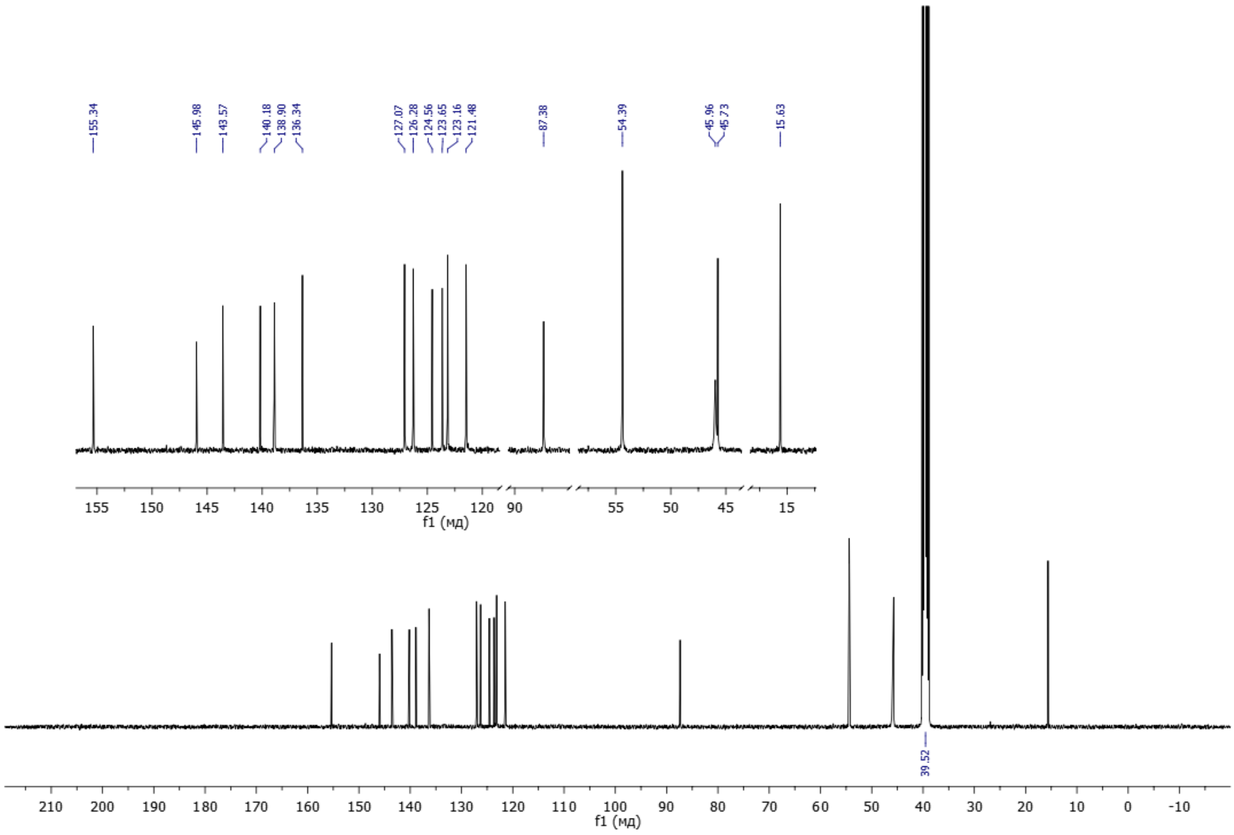

Figure S28. ^{13}C NMR spectrum of 2-methyl-4-(4-methylpiperazin-1-yl)-10-vinyl-10*H*-benzo[*b*]thieno[2,3-*e*][1,4]diazepine **(2n)**

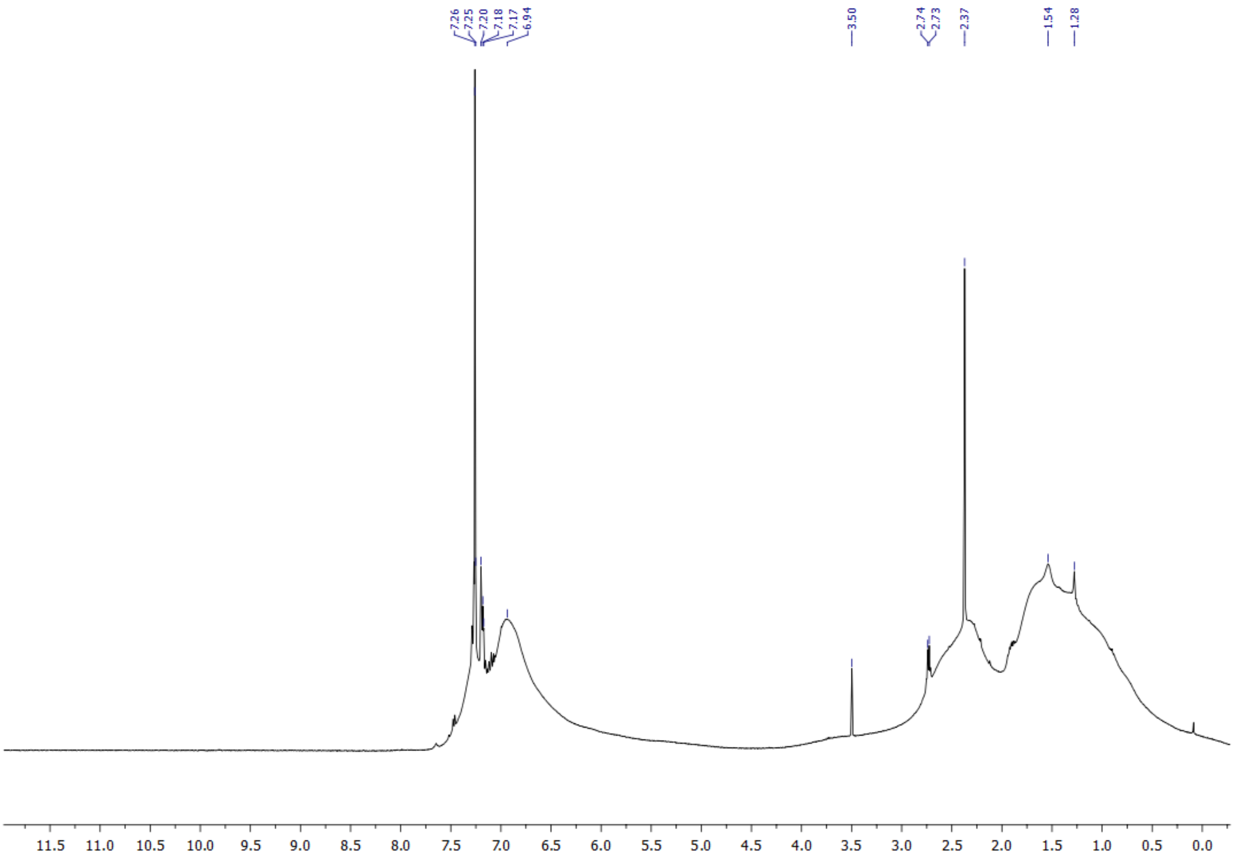

Figure S29. ^{1}H NMR spectrum of poly-*N*-vinyl-1,2,3,4-tetrahydrocarbazole **(3l)**

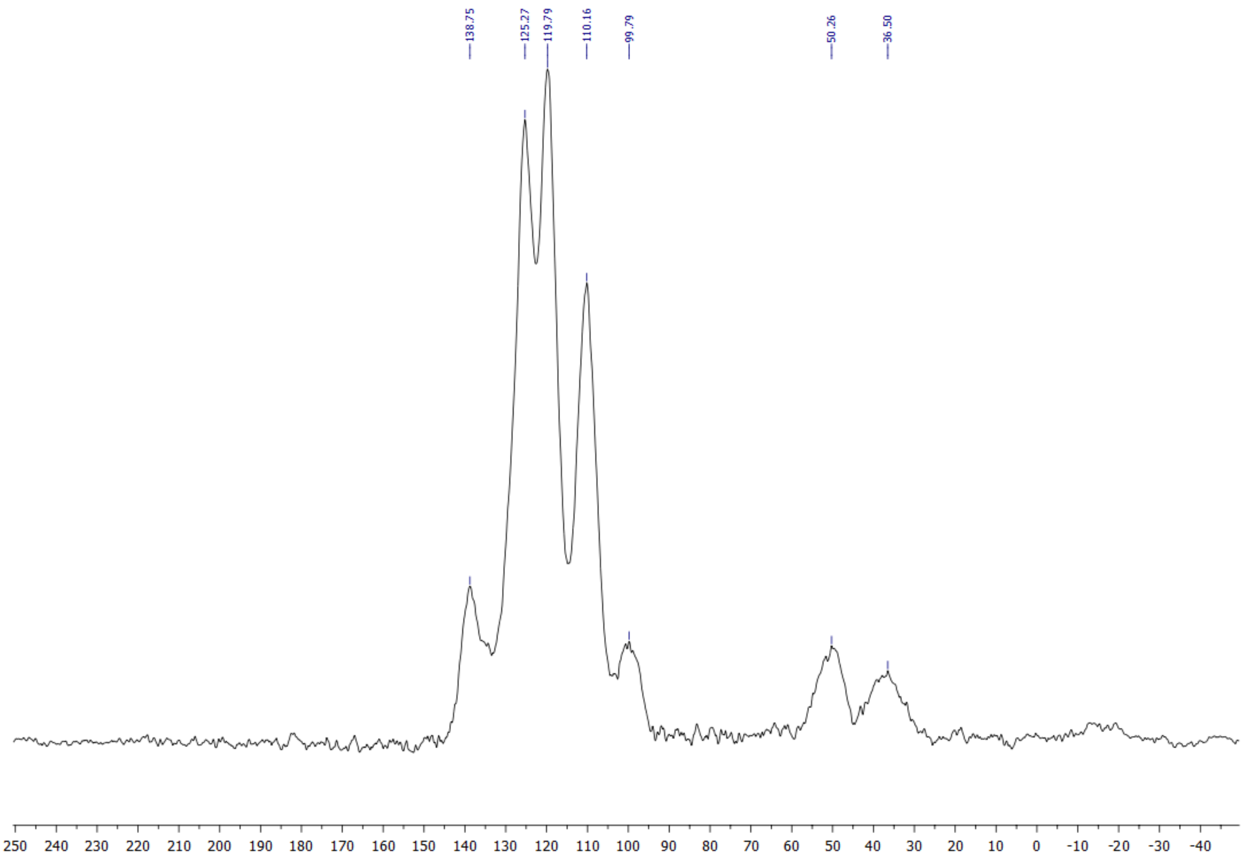

Figure S30. Solid state ^{13}C NMR spectrum of poly-*N*-vinyl-1,2,3,4-tetrahydrocarbazole **(3l)**

Crystal structures

5.1 X-ray crystallography data for 1-vinyl-1H-indole (2f)

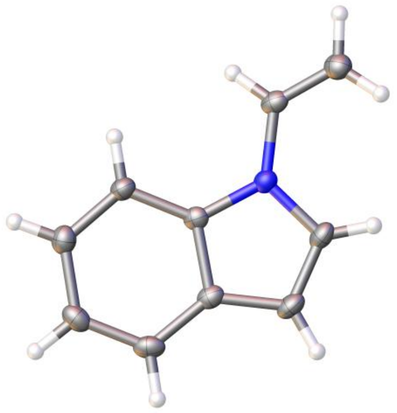

Figure S31. X-ray crystal structure of 1-vinyl-1*H*-indole (**2f**) (CCDC 1468160).

Table 1 Crystal data and structure refinement for **2f**

Empirical formula	$C_{10}H_9N$
Formula weight	143.18
Temperature/K	100(2)
Crystal system	orthorhombic
Space group	$Pna2_1$
a/Å	9.5037(17)
b/Å	5.9344(10)
c/Å	13.564(3)
α/°	90.00
β/°	90.00
γ/°	90.00
Volume/Å^3	765.0(2)
Z	4
ρ_{calc}mg/mm^3	1.243
m/mm^{-1}	0.073
F(000)	304.0
Crystal size/mm^3	0.31 × 0.09 × 0.08
2Θ range for data collection	6 to 55°
Index ranges	$-12 \le h \le 11$, $-7 \le k \le 7$, $-17 \le l \le 17$
Reflections collected	5207
Independent reflections	1682[R(int) = 0.0430]
Data/restraints/parameters	1682/1/100
Goodness-of-fit on F^2	1.027
Final R indexes [I>=2σ (I)]	R_1 = 0.0429, wR_2 = 0.1040

Final R indexes [all data]	R_1 = 0.0463, wR_2 = 0.1080
Largest diff. peak/hole / e Å^{-3}	0.20/-0.20
Flack parameter	-2(4)

Table 2 Fractional Atomic Coordinates (×10^4) and Equivalent Isotropic Displacement Parameters (Å^2×10^3) for . U_{eq} is defined as 1/3 of of the trace of the orthogonalised U_{IJ} tensor.

**Atom**	***x***	***y***	***z***	**U(eq)**
C5	8675.3(17)	9434(3)	7418.5(13)	18.3(4)
C6	8406.4(19)	7786(3)	8125.9(14)	20.1(4)
N1	9622.3(15)	9431(2)	6633.4(11)	19.1(3)
C8	6605.3(19)	10269(3)	8795.7(14)	23.8(4)
C7	7366(2)	8237(3)	8809.4(14)	23.5(4)
C2	7917.9(19)	11487(3)	7378.3(14)	19.9(4)
C3	8445(2)	12717(3)	6548.4(15)	23.2(4)
C10	11209(2)	7393(4)	5556.5(16)	25.6(4)
C9	10529.0(18)	7631(3)	6399.7(14)	20.8(4)
C1	6870.1(19)	11892(3)	8084.7(15)	23.3(4)
C4	9463(2)	11439(3)	6121.5(14)	22.2(4)

Table 3 Anisotropic Displacement Parameters (Å^2×10^3) for . The Anisotropic displacement factor exponent takes the form: $-2\pi^2[h^2a^{*2}U_{11}+...+2hka\times b\times U_{12}]$

**Atom**	**U_{11}**	**U_{22}**	**U_{33}**	**U_{23}**	**U_{13}**	**U_{12}**
C5	13.5(8)	18.1(9)	23.2(8)	-0.4(7)	-2.4(8)	-2.0(6)
C6	18.6(9)	17.5(8)	24.0(9)	1.6(7)	-1.7(7)	0.2(6)
N1	17.0(7)	17.7(7)	22.6(7)	2.4(6)	-0.8(6)	-0.6(6)
C8	16.7(9)	29.7(10)	24.8(9)	-6.7(8)	0.8(7)	-1.1(7)
C7	21.4(10)	25.7(10)	23.3(8)	2.0(7)	-1.1(8)	-4.3(7)
C2	17.7(9)	16.0(8)	26.1(9)	0.0(7)	-4.8(8)	-2.4(7)
C3	23.4(9)	16.2(8)	30.1(9)	4.5(8)	-5.5(8)	-2.1(6)
C10	23.4(10)	21.7(9)	31.7(9)	-0.1(7)	1.2(8)	-1.5(8)
C9	17.0(9)	16.2(8)	29.1(9)	0.8(7)	-3.7(7)	-1.9(6)
C1	16.8(9)	20.9(9)	32.2(10)	-4.5(8)	-5.6(8)	2.5(7)
C4	21.6(9)	18.5(9)	26.7(9)	6.2(7)	-3.8(7)	-4.0(7)

Table 4 Bond Lengths for **2f**.

**Atom**	**Atom**	**Length/Å**	**Atom**	**Atom**	**Length/Å**
C5	C6	1.394(3)	C8	C7	1.407(3)
C5	N1	1.394(2)	C8	C1	1.386(3)
C5	C2	1.416(3)	C2	C3	1.432(3)

C6	C7	1.381(3)	C2	C1	1.403(3)
N1	C9	1.408(2)	C3	C4	1.359(3)
N1	C4	1.388(2)	C10	C9	1.321(3)

Table 5 Bond Angles for **2f** .

**Atom**	**Atom**	**Atom**	**Angle/°**	**Atom**	**Atom**	**Atom**	**Angle/°**
C6	C5	N1	130.03(16)	C6	C7	C8	121.66(18)
C6	C5	C2	122.47(16)	C5	C2	C3	106.91(15)
N1	C5	C2	107.46(15)	C1	C2	C5	118.80(17)
C7	C6	C5	117.24(16)	C1	C2	C3	134.28(18)
C5	N1	C9	124.61(14)	C4	C3	C2	107.44(16)
C4	N1	C5	108.10(15)	C10	C9	N1	125.11(17)
C4	N1	C9	127.25(16)	C8	C1	C2	119.05(17)
C1	C8	C7	120.77(17)	C3	C4	N1	110.09(17)

Table 6 Hydrogen Atom Coordinates (Å×10^4) and Isotropic Displacement Parameters (Å^2×10^3) for **2f**.

**Atom**	***x***	***y***	***z***	**U(eq)**
H6	8906	6440	8137	24
H8	5916	10528	9269	29
H7	7164	7169	9291	28
H3	8145	14131	6340	28
H10A	11105	8465	5062	31
H10B	11790	6154	5458	31
H9	10660	6524	6876	25
H1	6360	13229	8076	28
H4	9979	11851	5567	27

Experimental

Crystals of $C_{10}H_9N$ were isolated at +11 °C, kept and transferred under cooling. A suitable crystal of $C_{10}H_9N$ was selected and placed on a diffractometer. The crystal was kept at 100(2) K during data collection. Using Olex2 [1], the structure was solved with the ShelXS [2] structure solution program using Direct Methods and refined with the ShelXL [3] refinement package using CGLS minimisation.

1. O. V. Dolomanov, L. J. Bourhis, R. J. Gildea, J. A. K. Howard and H. Puschmann, OLEX2: a complete structure solution, refinement and analysis program. J. Appl. Cryst. (2009). 42, 339-341.
2. SHELXS, G.M. Sheldrick, Acta Cryst. (2008). A64, 112-122
3. SHELXL, G.M. Sheldrick, Acta Cryst. (2008). A64, 112-122

Crystal Data. $C_{10}H_9N$, M =143.18, orthorhombic, a = 9.5037(17) Å, b = 5.9344(10) Å, c = 13.564(3) Å, V = 765.0(2) Å^3, T = 100(2), space group Pna2_1 (no. 33), Z = 4, μ(Mo Kα) = 0.073, 5207 reflections measured, 1682 unique (R_{int} = 0.0430) which were used in all calculations. The final wR_2 was 0.1080 (all data) and R_1 was 0.0429 (>2sigma(I)).

*5.2 X-ray crystallography data for 9,9'-divinyl-9H,9'H-3,3'-bicarbazole (**2m**)*

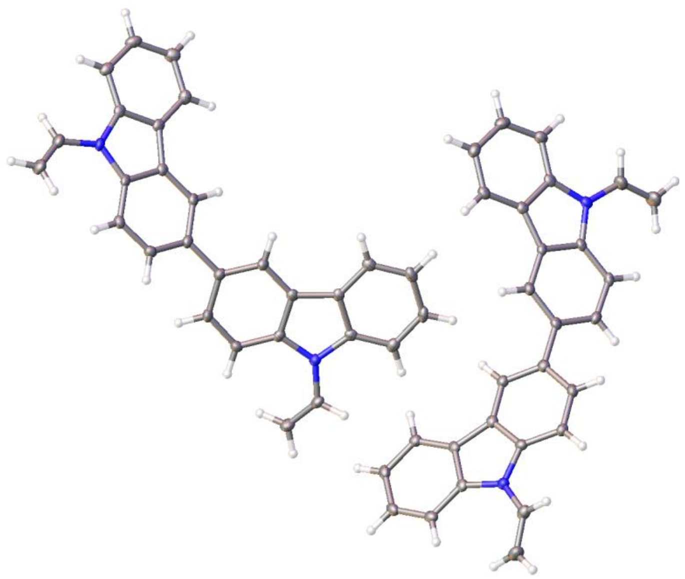

Figure S32. X-ray crystal structure of 9,9'-divinyl-9*H*,9'*H*-3,3'-bicarbazole (**2m**) (CCDC 1585616).

Crystal data for $C_{28}H_{20}N_2$ (M =384.46 g/mol): monoclinic, space group C2/c (no. 15), a = 60.6699(17) Å, b = 3.94085(11) Å, c = 32.2588(11) Å, β = 104.099(3)°, V = 7480.5(4) Å^3, Z = 16, T = 100.01(10) K, μ(MoKα) = 0.080 mm^{-1}, $Dcalc$ = 1.365 g/cm^3, 33586 reflections measured (6.468° ≤ 2Θ ≤ 55°), 8540 unique (R_{int} = 0.0468, R_{sigma} = 0.0438) which were used in all calculations. The final R_1 was 0.0758 (I > 2σ(I)) and wR_2 was 0.1683 (all data).

Table S1. Crystal data and structure refinement for 9,9'-divinyl-9*H*,9'*H*-3,3'-bicarbazole (**2m**)

Empirical formula	$C_{28}H_{20}N_2$
Formula weight	384.46
Temperature/K	100.01(10)
Crystal system	monoclinic
Space group	C2/c
a/Å	60.6699(17)
b/Å	3.94085(11)
c/Å	32.2588(11)
α/°	90
β/°	104.099(3)
γ/°	90
Volume/Å^3	7480.5(4)
Z	16
ρ_{calc}g/cm^3	1.365
μ/mm^{-1}	0.080

F(000)	3232.0
Crystal size/mm^3	0.2 × 0.2 × 0.15
Radiation	MoKα (λ = 0.71073)
2Θ range for data collection/°	6.468 to 55
Index ranges	-78 ≤ h ≤ 78, -5 ≤ k ≤ 5, -41 ≤ l ≤ 41
Reflections collected	33586
Independent reflections	8540 [R_{int} = 0.0468, R_{sigma} = 0.0438]
Data/restraints/parameters	8540/0/541
Goodness-of-fit on F^2	1.177
Final R indexes [I>=2σ (I)]	R_1 = 0.0758, wR_2 = 0.1622
Final R indexes [all data]	R_1 = 0.0902, wR_2 = 0.1683
Largest diff. peak/hole / e Å^{-3}	0.30/-0.34

Table S2. Fractional atomic coordinates (×10^4) and equivalent isotropic displacement parameters (Å^2×10^3) for 9,9'-divinyl-9*H*,9'*H*-3,3'-bicarbazole (**2m**). U_{eq} is defined as 1/3 of of the trace of the orthogonalised U_{IJ} tensor.

**Atom**	***x***	***y***	***z***	**U(eq)**
N1	5528.5(4)	4972(6)	6675.1(7)	16.6(5)
N2	6690.8(4)	1796(6)	5006.3(7)	15.7(5)
N3	8111.0(4)	9397(6)	6976.0(7)	16.1(5)
N4	5503.7(4)	10476(6)	3682.0(7)	17.0(5)
C5	5664.2(4)	8318(7)	4347.4(8)	16.3(5)
C6	7113.1(4)	2868(7)	5092.6(8)	17.4(5)
C7	7095.5(4)	5593(7)	5892.0(8)	16.6(5)
C8	5489.2(4)	7282(7)	5390.0(8)	16.7(5)
C9	5282.7(4)	10597(7)	4253.1(8)	17.2(5)
C10	7553.9(4)	6013(7)	6511.2(8)	16.1(5)
C11	5842.5(4)	7601(7)	6547.0(8)	16.3(5)
C12	7845.9(4)	6637(7)	7245.0(8)	16.9(5)
C13	5293.9(4)	9686(7)	4671.7(8)	18.6(6)
C14	6909.0(4)	2899(7)	5218.4(8)	15.2(5)
C15	6899.6(4)	4359(7)	5612.8(8)	15.2(5)
C16	6550.1(5)	5295(7)	5941.5(8)	18.4(5)
C17	7923.4(4)	8736(7)	6629.8(8)	15.3(5)
C18	6201.4(5)	3046(7)	5497.7(9)	20.2(6)
C19	7516.0(4)	6631(7)	6074.4(8)	16.4(5)
C20	6042.1(5)	7567(8)	3421.5(8)	20.5(6)
C21	5472.4(4)	9900(7)	4093.9(8)	15.9(5)
C22	7756.6(4)	7033(7)	6789.2(8)	15.6(5)
C23	7304.5(4)	4175(7)	5370.1(8)	18.4(6)
C24	5678.7(4)	7122(7)	6141.1(8)	15.1(5)
C25	6145.4(5)	6199(7)	3819.7(9)	20.1(6)
C26	5673.5(4)	7483(7)	4772.8(8)	16.9(5)

C27	6664.3(4)	4210(7)	5637.9(8)	15.3(5)
C28	6318.9(5)	4693(7)	5867.4(9)	21.1(6)
C29	7889.1(4)	9371(7)	6195.6(8)	17.2(5)
C30	6058.5(5)	9045(7)	6660.0(8)	18.7(6)
C31	7758.6(5)	5219(7)	7567.4(9)	19.5(6)
C32	6034.8(4)	6319(7)	4151.7(8)	17.1(5)
C33	5298.5(4)	5786(7)	5491.8(8)	17.9(5)
C34	6542.7(4)	2597(7)	5263.3(8)	15.4(5)
C35	6311.2(5)	1956(7)	5189.4(9)	19.1(6)
C36	5717.7(4)	9142(7)	3670.1(8)	16.3(5)
C37	5485.8(4)	8167(7)	4939.1(8)	17.4(5)
C38	5294.1(4)	4865(7)	5903.8(8)	17.3(5)
C39	6618.0(5)	258(7)	4606.1(8)	18.2(6)
C40	8063.5(4)	8104(7)	7347.4(8)	16.6(5)
C41	5746.0(4)	6251(7)	6864.1(8)	17.3(5)
C42	5820.9(4)	7796(7)	4076.8(8)	16.1(5)
C43	7302.4(4)	5478(7)	5774.9(8)	16.4(5)
C44	5486.6(4)	5526(7)	6231.9(8)	15.6(5)
C45	7687.0(4)	8293(7)	5925.5(8)	17.7(5)
C46	5679.5(4)	7929(7)	5721.6(8)	17.1(5)
C47	5861.0(5)	6269(7)	7294.6(8)	19.8(6)
C48	8196.1(5)	8183(7)	7767.9(8)	20.1(6)
C49	5828.5(5)	9055(7)	3338.9(8)	19.7(6)
C50	6741.5(5)	-1328(8)	4385.3(9)	23.5(6)
C51	7888.2(5)	5313(8)	7985.3(9)	22.9(6)
C52	8104.4(5)	6761(7)	8080.8(9)	22.9(6)
C53	6076.1(5)	7670(8)	7399.6(9)	23.3(6)
C54	5347.9(5)	12225(8)	3368.0(9)	22.0(6)
C55	6175.1(5)	9057(8)	7089.2(9)	21.6(6)
C56	5324.5(5)	12173(9)	2947.6(9)	29.1(7)
C57	8323.9(5)	10751(8)	6967.3(9)	22.1(6)
C58	8376.8(5)	12606(8)	6666.9(9)	24.4(6)
C59	5386.3(5)	3655(8)	6913.6(9)	24.3(6)
C60	5198.5(5)	1928(9)	6790.4(10)	29.6(7)

Table S3. Anisotropic displacement parameters (Å^2×10^3) for 9,9'-divinyl-9*H*,9'*H*-3,3'-bicarbazole (**2m**). The anisotropic displacement factor exponent takes the form: $-2\pi^2[h^2a^{*2}U_{11}+2hka^*b^*U_{12}+\ldots]$.

**Atom**	**U_{11}**	**U_{22}**	**U_{33}**	**U_{23}**	**U_{13}**	**U_{12}**
N1	17.3(11)	17.7(11)	16.1(10)	-0.7(9)	6.5(8)	2.5(9)
N2	14.6(10)	16.7(11)	15.7(10)	-0.4(9)	3.2(8)	-1.2(9)
N3	10.2(10)	18.0(11)	19.7(11)	-1.7(9)	2.7(8)	0.0(9)
N4	15.4(11)	20.2(12)	15(1)	0.2(9)	3.0(8)	-1.9(9)

C5	15.4(12)	15.1(13)	18.4(12)	-2.5(10)	3.8(10)	-1.6(11)
C6	17.3(13)	21.3(14)	14.0(12)	-1.6(11)	4.8(10)	2.0(11)
C7	14.6(12)	17.3(13)	17.5(12)	-1(1)	3.1(10)	2.2(11)
C8	15.9(12)	16.6(13)	18.6(12)	-1.8(10)	6.2(10)	2.2(11)
C9	14.4(12)	17.4(13)	18.5(12)	-1.3(11)	1.6(10)	1.1(11)
C10	12.1(12)	16.4(13)	20.4(12)	-1.8(11)	5.3(10)	1.4(10)
C11	18.0(13)	14.4(13)	17.5(12)	-1.5(10)	5.9(10)	2.3(11)
C12	15.2(12)	15.1(13)	19.3(12)	-2.4(10)	2.3(10)	4.3(10)
C13	15.0(13)	20.6(14)	21.6(13)	-3.9(11)	7.1(10)	-0.2(11)
C14	14.6(12)	13.1(12)	16.4(12)	2.7(10)	0.7(10)	1(1)
C15	16.3(12)	13.8(12)	15.2(12)	2.4(10)	3.5(10)	2.9(10)
C16	22.3(14)	14.4(13)	18.9(12)	2.3(10)	6.2(10)	2.0(11)
C17	10.8(11)	15.4(13)	19.1(12)	-4.1(10)	2.5(9)	2.6(10)
C18	12.6(12)	19.8(14)	29.0(14)	6.6(12)	6.4(11)	0.2(11)
C19	11.8(12)	16.7(13)	20.4(13)	-2.3(11)	3(1)	1.9(10)
C20	21.1(14)	23.8(15)	18.7(13)	-3.9(11)	9.1(11)	-7.4(12)
C21	16.9(13)	16.3(13)	14.2(12)	-0.9(10)	2.8(10)	-2.5(11)
C22	14.5(12)	14.0(12)	18.8(12)	-1.5(10)	4.8(10)	2.5(10)
C23	12.5(12)	23.0(14)	20.1(13)	1.1(11)	4.4(10)	2.5(11)
C24	11.5(11)	14.4(12)	20.0(12)	-3.5(10)	5(1)	0.2(10)
C25	15.8(13)	21.5(14)	23.9(13)	-5.3(11)	6.8(11)	-4.2(11)
C26	15.0(12)	18.0(13)	16.9(12)	-1.0(11)	2.6(10)	-1.0(11)
C27	15.8(12)	12.9(12)	17.2(12)	4.2(10)	4.1(10)	0.6(10)
C28	21.5(14)	20.8(14)	24.3(14)	5.5(12)	11.6(11)	5.8(12)
C29	15.7(12)	17.0(13)	20.7(13)	-1.5(11)	7.8(10)	0.0(11)
C30	19.6(13)	18.4(14)	20.2(13)	-1.8(11)	8.7(10)	1.7(11)
C31	18.0(13)	16.2(13)	24.2(13)	1.7(11)	4.7(11)	0.6(11)
C32	16.3(12)	17.3(13)	17.3(12)	-2.4(10)	3.1(10)	-2.1(11)
C33	12.5(12)	20.0(14)	20.0(12)	-3.6(11)	1.6(10)	0.2(11)
C34	18.5(13)	12.4(12)	14.9(12)	3.2(10)	3.4(10)	2(1)
C35	18.5(13)	16.4(13)	20.9(13)	3.3(11)	2(1)	0.2(11)
C36	14.7(12)	14.7(13)	19.5(12)	-3.2(10)	4.3(10)	-4.6(10)
C37	17.0(13)	17.4(13)	17.8(12)	-2.7(11)	4.2(10)	-2.1(11)
C38	13.2(12)	16.7(13)	23.4(13)	-0.7(11)	7.4(10)	-1.3(10)
C39	16.1(13)	19.5(14)	17.2(12)	0.1(11)	0.2(10)	-3.7(11)
C40	15.0(12)	13.9(12)	20.6(13)	-1.9(10)	3.7(10)	3.5(10)
C41	18.8(13)	14.4(13)	20.0(13)	-1.5(10)	7.5(10)	4.7(11)
C42	17.0(12)	15.4(13)	16.6(12)	-2.9(10)	5.6(10)	-5.1(11)
C43	14.8(12)	16.2(13)	16.5(12)	-0.5(10)	0.8(10)	1.6(11)
C44	15.4(12)	15.6(13)	17.9(12)	-0.3(10)	8.2(10)	3.1(11)
C45	18.5(13)	20.1(14)	14.6(12)	0.3(10)	4.2(10)	2.6(11)
C46	14.6(12)	18.1(13)	20.1(12)	-2.8(11)	7(1)	-0.6(11)
C47	21.7(14)	21.7(14)	16.9(12)	1.7(11)	6.3(10)	3.6(12)

C48	19.8(13)	16.2(13)	21.7(13)	-3.7(11)	-0.1(11)	1.5(11)
C49	21.9(14)	21.1(14)	16.3(12)	-3.0(11)	4.7(10)	-6.2(12)
C50	21.9(14)	26.7(16)	20.4(13)	-6.0(12)	2.2(11)	-3.2(13)
C51	29.0(15)	18.4(14)	21.2(13)	2.0(11)	5.9(11)	-0.4(12)
C52	27.7(15)	19.7(14)	17.6(13)	-1.4(11)	-1.5(11)	2.1(12)
C53	25.1(15)	23.8(15)	18.7(13)	-4.1(12)	0.8(11)	3.1(12)
C54	18.5(13)	22.3(15)	23.5(14)	3.9(12)	1.9(11)	-2.8(12)
C55	16.8(13)	22.1(14)	25.5(14)	-4.4(12)	4.1(11)	-1.4(12)
C56	23.6(15)	38.9(19)	22.1(14)	6.3(13)	0.5(12)	-0.4(14)
C57	15.4(13)	25.7(15)	23.7(14)	-3.4(12)	2.1(11)	-2.6(12)
C58	18.6(14)	27.1(16)	25.5(14)	-2.1(12)	1.8(11)	-6.1(12)
C59	23.5(14)	30.6(16)	20.6(13)	3.7(12)	9.2(11)	-0.4(13)
C60	28.7(16)	37.3(18)	24.6(15)	5.4(14)	9.9(12)	-7.5(14)

Table S4. Bond lengths for 9,9'-divinyl-9*H*,9'*H*-3,3'-bicarbazole (**2m**).

Atom	Atom	Length/Å	Atom	Atom	Length/Å
N1	C41	1.406(3)	C16	C27	1.397(4)
N1	C44	1.407(3)	C16	C28	1.385(4)
N1	C59	1.389(3)	C17	C22	1.411(4)
N2	C14	1.403(3)	C17	C29	1.388(4)
N2	C34	1.399(3)	C18	C28	1.392(4)
N2	C39	1.397(3)	C18	C35	1.393(4)
N3	C17	1.411(3)	C19	C43	1.486(3)
N3	C40	1.395(3)	C19	C45	1.407(4)
N3	C57	1.404(3)	C20	C25	1.393(4)
N4	C21	1.406(3)	C20	C49	1.388(4)
N4	C36	1.409(3)	C23	C43	1.406(4)
N4	C54	1.389(3)	C24	C44	1.416(4)
C5	C21	1.396(4)	C24	C46	1.391(4)
C5	C26	1.399(4)	C25	C32	1.396(4)
C5	C42	1.453(4)	C26	C37	1.398(4)
C6	C14	1.394(4)	C27	C34	1.405(4)
C6	C23	1.382(4)	C29	C45	1.386(4)
C7	C15	1.393(4)	C30	C55	1.393(4)
C7	C43	1.397(4)	C31	C51	1.386(4)
C8	C33	1.407(4)	C32	C42	1.389(4)
C8	C37	1.491(4)	C33	C38	1.384(4)
C8	C46	1.393(4)	C34	C35	1.390(4)
C9	C13	1.383(4)	C36	C42	1.412(4)
C9	C21	1.397(4)	C36	C49	1.394(4)
C10	C19	1.393(4)	C38	C44	1.396(4)
C10	C22	1.393(4)	C39	C50	1.312(4)
C11	C24	1.450(4)	C40	C48	1.399(4)

C11	C30	1.393(4)	C41	C47	1.394(4)
C11	C41	1.401(4)	C47	C53	1.381(4)
C12	C22	1.446(4)	C48	C52	1.384(4)
C12	C31	1.394(4)	C51	C52	1.395(4)
C12	C40	1.405(4)	C53	C55	1.398(4)
C13	C37	1.403(4)	C54	C56	1.329(4)
C14	C15	1.410(4)	C57	C58	1.315(4)
C15	C27	1.450(4)	C59	C60	1.303(4)

Table S5. Bond angles for 9,9'-divinyl-9*H*,9'*H*-3,3'-bicarbazole (**2m**).

Atom	Atom	Atom	Angle/°	Atom	Atom	Atom	Angle/°
C41	N1	C44	107.6(2)	C46	C24	C11	133.2(2)
C59	N1	C41	122.6(2)	C46	C24	C44	120.0(2)
C59	N1	C44	129.7(2)	C20	C25	C32	120.1(3)
C34	N2	C14	107.9(2)	C37	C26	C5	119.3(2)
C39	N2	C14	129.3(2)	C16	C27	C15	133.6(2)
C39	N2	C34	122.8(2)	C16	C27	C34	119.7(2)
C40	N3	C17	108.5(2)	C34	C27	C15	106.7(2)
C40	N3	C57	122.6(2)	C16	C28	C18	120.9(3)
C57	N3	C17	128.5(2)	C45	C29	C17	118.1(2)
C21	N4	C36	107.6(2)	C55	C30	C11	118.6(3)
C54	N4	C21	123.0(2)	C51	C31	C12	119.1(3)
C54	N4	C36	129.3(2)	C42	C32	C25	119.0(2)
C21	C5	C26	120.6(2)	C38	C33	C8	122.9(2)
C21	C5	C42	106.6(2)	N2	C34	C27	109.5(2)
C26	C5	C42	132.8(2)	C35	C34	N2	128.7(2)
C23	C6	C14	118.3(2)	C35	C34	C27	121.8(2)
C15	C7	C43	119.7(2)	C34	C35	C18	117.4(3)
C33	C8	C37	120.6(2)	N4	C36	C42	108.5(2)
C46	C8	C33	118.2(2)	C49	C36	N4	130.5(2)
C46	C8	C37	121.2(2)	C49	C36	C42	121.0(2)
C13	C9	C21	117.3(2)	C13	C37	C8	120.8(2)
C19	C10	C22	120.2(2)	C26	C37	C8	120.7(2)
C30	C11	C24	133.1(2)	C26	C37	C13	118.5(2)
C30	C11	C41	119.8(2)	C33	C38	C44	118.1(2)
C41	C11	C24	107.1(2)	C50	C39	N2	127.9(3)
C31	C12	C22	133.3(3)	N3	C40	C12	109.1(2)
C31	C12	C40	119.7(2)	N3	C40	C48	129.4(3)
C40	C12	C22	107.0(2)	C48	C40	C12	121.5(3)
C9	C13	C37	123.2(2)	C11	C41	N1	109.5(2)
N2	C14	C15	109.0(2)	C47	C41	N1	128.6(2)
C6	C14	N2	130.9(2)	C47	C41	C11	121.9(3)
C6	C14	C15	120.0(2)	C32	C42	C5	132.5(2)

C7	C15	C14	120.7(2)	C32	C42	C36	120.2(2)
C7	C15	C27	132.4(2)	C36	C42	C5	107.3(2)
C14	C15	C27	106.9(2)	C7	C43	C19	120.9(2)
C28	C16	C27	118.7(3)	C7	C43	C23	118.3(2)
N3	C17	C22	108.1(2)	C23	C43	C19	120.8(2)
C29	C17	N3	131.2(2)	N1	C44	C24	109.0(2)
C29	C17	C22	120.6(2)	C38	C44	N1	130.7(2)
C28	C18	C35	121.4(3)	C38	C44	C24	120.3(2)
C10	C19	C43	120.5(2)	C29	C45	C19	122.7(2)
C10	C19	C45	118.2(2)	C24	C46	C8	120.4(2)
C45	C19	C43	121.3(2)	C53	C47	C41	117.4(3)
C49	C20	C25	122.0(2)	C52	C48	C40	117.4(3)
C5	C21	N4	109.9(2)	C20	C49	C36	117.7(3)
C5	C21	C9	121.1(2)	C31	C51	C52	120.4(3)
C9	C21	N4	129.0(2)	C48	C52	C51	121.9(3)
C10	C22	C12	132.6(2)	C47	C53	C55	121.7(3)
C10	C22	C17	120.1(2)	C56	C54	N4	128.4(3)
C17	C22	C12	107.3(2)	C30	C55	C53	120.5(3)
C6	C23	C43	122.8(2)	C58	C57	N3	128.1(3)
C44	C24	C11	106.8(2)	C60	C59	N1	129.9(3)

Table S6. Hydrogen atom coordinates (Å×10^4) and isotropic displacement parameters (Å^2×10^3) for 9,9'-divinyl-9*H*,9'*H*-3,3'-bicarbazole (**2m**).

**Atom**	***x***	***y***	***z***	**U(eq)**
H6	7120	1989	4829	21
H7	7089	6489	6155	20
H9	5154	11632	4084	21
H10	7444	4914	6618	19
H13	5168	10101	4781	22
H16	6628	6401	6189	22
H18	6046	2664	5456	24
H20	6119	7481	3205	25
H23	7440	4194	5286	22
H25	6288	5203	3864	24
H26	5803	6484	4943	20
H28	6241	5399	6067	25
H29	7999	10488	6089	21
H30	6123	9982	6453	22
H31	7615	4224	7503	23
H32	6103	5425	4419	21
H33	5170	5399	5273	21

H35	6233	843	4944	23
H38	5167	3835	5960	21
H39	6463	372	4481	22
H45	7664	8686	5634	21
H46	5808	8906	5662	21
H47	5795	5372	7503	24
H48	8340	9153	7835	24
H49	5762	9963	3072	24
H50A	6898	-1525	4494	28
H50B	6673	-2256	4120	28
H51	7831	4404	8203	27
H52	8189	6772	8363	27
H53	6157	7692	7684	28
H54	5247	13613	3464	26
H55	6320	9994	7170	26
H56A	5420	10834	2831	35
H56B	5212	13474	2770	35
H57	8442	10276	7204	26
H58A	8266	13166	6423	29
H58B	8525	13358	6699	29
H59	5432	4069	7206	29
H60A	5142	1416	6503	36
H60B	5121	1209	6990	36

Experimental

The crystal was kept at 100.01(10) K during data collection. Using Olex2 [1], the structure was solved with the Superflip [2] structure solution program using Charge Flipping and refined with the ShelXL [3] refinement package using Least Squares minimisation.

1. Dolomanov, O.V., Bourhis, L.J., Gildea, R.J, Howard, J.A.K. & Puschmann, H. (2009), J. Appl. Cryst. 42, 339-341.
2. Palatinus, L. & Chapuis, G. (2007). J. Appl. Cryst., 40, 786-790; Palatinus, L. & van der Lee, A. (2008). J. Appl. Cryst. 41, 975-984; Palatinus, L., Prathapa, S. J. & van Smaalen, S. (2012). J. Appl. Cryst. 45, 575-580.
3. Sheldrick, G.M. (2015). Acta Cryst. C71, 3-8.

5.3 X-ray crystallography data for 2-methyl-4-(4-methylpiperazin-1-yl)-10-vinyl-10H-benzo[b]thieno[2,3-e][1,4]diazepine ***(2n)***

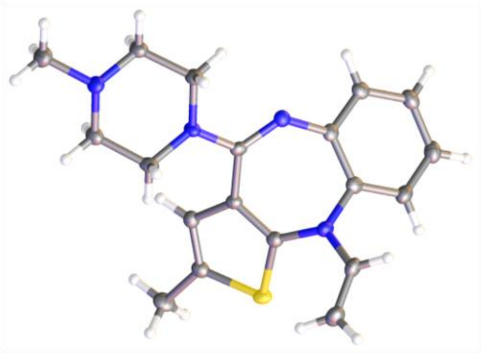

Figure S33. X-ray crystal structure of 2-methyl-4-(4-methylpiperazin-1-yl)-10-vinyl-10*H*-benzo[*b*]thieno[2,3-*e*][1,4]diazepine **(2n)** (CCDC 1585614).

Crystal data for $C_{19}H_{22}N_4S$ (M =338.46 g/mol): monoclinic, space group P2$_1$/n (no. 14), a = 8.9450(4) Å, b = 14.1199(8) Å, c = 14.0081(6) Å, β = 98.512(4)°, V = 1749.77(15) Å^3, Z = 4, T = 100.00(10) K, μ(MoKα) = 0.193 mm^{-1}, $Dcalc$ = 1.285 g/cm^3, 8032 reflections measured (5.084° ≤ 2Θ ≤ 55°), 4001 unique (R_{int} = 0.0306, R_{sigma} = 0.0594) which were used in all calculations. The final R_1 was 0.0488 (I > 2σ(I)) and wR_2 was 0.1070 (all data).

Table S7. Crystal data and structure refinement for 2-methyl-4-(4-methylpiperazin-1-yl)-10-vinyl-10*H*-benzo[*b*]thieno[2,3-*e*][1,4]diazepine **(2n)**

Empirical formula	$C_{19}H_{22}N_4S$
Formula weight	338.46
Temperature/K	100.00(10)
Crystal system	monoclinic
Space group	P2$_1$/n
a/Å	8.9450(4)
b/Å	14.1199(8)
c/Å	14.0081(6)
α/°	90
β/°	98.512(4)
γ/°	90
Volume/Å^3	1749.77(15)
Z	4
ρ_{calc}g/cm^3	1.285
μ/mm^{-1}	0.193
F(000)	720.0
Crystal size/mm^3	0.15 × 0.15 × 0.1
Radiation	MoKα (λ = 0.71073)
2Θ range for data collection/°	5.084 to 55
Index ranges	-11 ≤ h ≤ 11, -18 ≤ k ≤ 7, -12 ≤ l ≤ 18
Reflections collected	8032
Independent reflections	4001 [R_{int} = 0.0306, R_{sigma} = 0.0594]
Data/restraints/parameters	4001/0/219
Goodness-of-fit on F^2	1.027
Final R indexes [I>=2σ (I)]	R_1 = 0.0488, wR_2 = 0.0948

Final R indexes [all data]	R_1 = 0.0786, wR_2 = 0.1070
Largest diff. peak/hole / e Å^{-3}	0.40/-0.27

Table S8. Fractional atomic coordinates (×10^4) and equivalent isotropic displacement parameters (Å^2×10^3) for 2-methyl-4-(4-methylpiperazin-1-yl)-10-vinyl-10*H*-benzo[*b*]thieno[2,3-*e*][1,4]-diazepine **(2n)**. U_{eq} is defined as 1/3 of of the trace of the orthogonalised U_{IJ} tensor.

**Atom**	***x***	***y***	***z***	**U(eq)**
S1	7240.3(6)	7893.1(4)	3143.2(3)	20.97(14)
N1	10227.3(17)	8404.1(12)	6125.9(10)	17.4(4)
N2	8457.0(17)	9542.3(13)	6283.4(10)	18.4(4)
N3	7398.7(18)	11015.1(13)	7408.0(11)	21.2(4)
C4	8038(2)	7857.5(15)	4353.0(12)	17.5(4)
N5	8619.9(18)	7030.1(12)	4822.4(11)	20.1(4)
C6	12545(2)	7708.0(15)	5810.3(13)	19.7(4)
C7	10963(2)	7734.7(14)	5626.9(12)	17.4(4)
C8	9169(2)	9706.5(15)	7278.6(13)	21.9(5)
C9	7793(2)	9463.5(15)	4042.5(12)	18.8(4)
C10	6404(2)	6038.0(17)	4404.3(14)	26.7(5)
C11	9026(2)	8856.3(14)	5745.9(12)	16.5(4)
C12	6513(2)	9654.8(17)	2285.1(13)	26.1(5)
C13	6870(2)	9833.1(16)	6129.6(13)	21.3(5)
C14	7825(2)	6183.1(15)	4788.6(14)	22.4(4)
C15	13370(2)	7046.1(16)	5384.1(13)	22.9(5)
C16	8269(2)	8737.5(14)	4736.7(12)	15.9(4)
C17	10236(2)	7030.5(15)	5012.9(13)	18.6(4)
C18	12629(2)	6370.9(16)	4764.5(14)	24.9(5)
C19	8984(2)	10730.3(16)	7545.5(13)	22.1(5)
C1A	6755(2)	10866.5(16)	6392.1(13)	22.6(5)
C1B	7204(2)	9121.2(15)	3162.2(13)	19.9(4)
C1C	11066(2)	6371.3(15)	4580.8(13)	22.6(5)
C1D	7264(3)	12010.6(17)	7674.6(15)	29.8(5)

Table S9. Anisotropic displacement parameters (Å^2×10^3) for 2-methyl-4-(4-methylpiperazin-1-yl)-10-vinyl-10*H*-benzo[*b*]thieno[2,3-*e*][1,4]-diazepine **(2n)**. The anisotropic displacement factor exponent takes the form: $-2\pi^2[h^2a^{*2}U_{11}+2hka^*b^*U_{12}+\ldots]$.

**Atom**	**U_{11}**	**U_{22}**	**U_{33}**	**U_{23}**	**U_{13}**	**U_{12}**
S1	21.7(3)	24.6(3)	15.9(2)	-4.0(2)	0.21(17)	-0.8(2)
N1	19.2(8)	17.3(10)	16.0(7)	1.9(7)	3.2(6)	-0.5(7)
N2	18.6(8)	22.2(10)	13.6(7)	-2.9(7)	-0.8(6)	2.7(7)
N3	21.5(9)	23.7(10)	18.3(8)	-4.7(7)	2.5(6)	3.6(8)
C4	16.4(9)	20.0(11)	16.4(9)	0.1(8)	3.0(7)	-0.9(9)
N5	21.5(9)	16.3(10)	21.3(8)	-1.1(7)	-0.6(6)	-1.7(7)
C6	21.8(10)	20.0(12)	16.8(9)	4.6(8)	1.9(7)	0.2(9)
C7	21.9(10)	16.7(11)	14.1(8)	4.0(8)	4.0(7)	1.7(8)
C8	24.1(10)	24.6(12)	15.4(9)	-3.8(9)	-2.4(7)	3.3(9)
C9	18.6(10)	18.2(11)	19.9(9)	1.3(8)	3.7(7)	0.7(9)
C10	26.0(11)	20.5(12)	34.5(11)	-3.1(10)	6.9(9)	-4.4(10)

C11	17.5(10)	15.1(10)	16.5(9)	0.1(8)	1.6(7)	-4.5(8)
C12	27.7(11)	32.5(14)	17.0(9)	5.6(9)	0.1(8)	-0.4(10)
C13	15.9(9)	29.8(13)	17.8(9)	-3.3(9)	0.6(7)	-0.3(9)
C14	27.3(11)	15.2(11)	25.3(10)	-0.7(9)	6.2(8)	-0.6(9)
C15	20.1(10)	26.6(13)	22.2(10)	5.7(9)	3.5(8)	4.4(9)
C16	14.0(9)	18.8(11)	15.1(9)	-1.1(8)	2.6(7)	-1.3(8)
C17	21.5(10)	16.4(11)	17.7(9)	4.3(8)	2.3(7)	-1.5(9)
C18	29.2(11)	23.9(13)	22.7(10)	1.8(9)	7.9(8)	7.5(10)
C19	21.1(10)	25.7(12)	18.8(9)	-4.8(9)	0.4(7)	0.4(9)
C1A	19.4(10)	27.9(13)	20.5(9)	-2.5(9)	2.7(7)	4.1(9)
C1B	17.1(9)	24.4(12)	18.2(9)	0.8(9)	3.1(7)	0.3(9)
C1C	29.7(11)	17.9(12)	19.8(9)	-1.4(9)	2.3(8)	1.3(9)
C1D	30.0(12)	28.8(14)	30.2(11)	-8(1)	3.5(9)	5.1(11)

Table S10. Bond lengths for 2-methyl-4-(4-methylpiperazin-1-yl)-10 10*H*-benzo[*b*]thieno[2,3-*e*][1,4]-diazepine **(2n)**.

Atom	Atom	Length/Å	Atom	Atom	Length/Å
S1	C4	1.7396(18)	C6	C7	1.401(3)
S1	C1B	1.735(2)	C6	C15	1.380(3)
N1	C7	1.397(2)	C7	C17	1.409(3)
N1	C11	1.295(2)	C8	C19	1.508(3)
N2	C8	1.463(2)	C9	C16	1.434(3)
N2	C11	1.370(2)	C9	C1B	1.356(3)
N2	C13	1.463(2)	C10	C14	1.321(3)
N3	C19	1.459(2)	C11	C16	1.484(2)
N3	C1A	1.469(2)	C12	C1B	1.495(3)
N3	C1D	1.464(3)	C13	C1A	1.512(3)
C4	N5	1.402(3)	C15	C18	1.390(3)
C4	C16	1.357(3)	C17	C1C	1.385(3)
N5	C14	1.389(3)	C18	C1C	1.383(3)
N5	C17	1.430(2)			

Table S11. Bond angles for 2-methyl-4-(4-methylpiperazin-1-yl)-10-vinyl-10*H*-benzo[*b*]thieno[2,3-*e*][1,4]-diazepine **(2n)**.

Atom	Atom	Atom	Angle/°	Atom	Atom	Atom	Angle/°
C1B	S1	C4	91.14(9)	N1	C11	N2	118.42(16)
C11	N1	C7	123.49(16)	N1	C11	C16	124.76(17)
C8	N2	C13	111.83(14)	N2	C11	C16	116.66(17)
C11	N2	C8	119.40(16)	N2	C13	C1A	109.54(16)
C11	N2	C13	123.29(15)	C10	C14	N5	127.2(2)
C19	N3	C1A	108.81(14)	C6	C15	C18	119.92(19)
C19	N3	C1D	110.26(16)	C4	C16	C9	111.93(16)
C1D	N3	C1A	110.42(16)	C4	C16	C11	120.12(17)
N5	C4	S1	123.45(15)	C9	C16	C11	127.83(18)
C16	C4	S1	112.06(15)	C7	C17	N5	118.47(18)
C16	C4	N5	123.57(16)	C1C	C17	N5	120.72(18)
C4	N5	C17	112.43(16)	C1C	C17	C7	120.78(18)

C14	N5	C4	123.30(16)	C1C	C18	C15	119.42(19)
C14	N5	C17	120.32(17)	N3	C19	C8	111.82(17)
C15	C6	C7	121.94(19)	N3	C1A	C13	110.02(17)
N1	C7	C6	117.68(17)	C9	C1B	S1	111.38(15)
N1	C7	C17	124.99(17)	C9	C1B	C12	128.7(2)
C6	C7	C17	117.10(18)	C12	C1B	S1	119.85(15)
N2	C8	C19	109.86(16)	C18	C1C	C17	120.78(19)
C1B	C9	C16	113.47(19)				

Table S12. Hydrogen atom coordinates (Å×10^4) and isotropic displacement parameters (Å^2×10^3) for 2-methyl-4-(4-methylpiperazin-1-yl)-10-vinyl-10*H*-benzo[*b*]thieno[2,3-*e*][1,4]-diazepine **(2n)**.

**Atom**	***x***	***y***	***z***	**U(eq)**
H6	13055	8149	6232	24
H8A	10235	9550	7344	26
H8B	8706	9302	7712	26
H9	7879	10107	4182	23
H10A	5825	6537	4116	32
H10B	5983	5437	4422	32
H12A	6943	9439	1735	39
H12B	6710	10319	2380	39
H12C	5441	9549	2177	39
H13A	6302	9451	6526	26
H13B	6443	9738	5459	26
H14	8351	5660	5067	27
H15	14420	7052	5511	28
H18	13179	5923	4476	30
H19A	9415	10825	8216	27
H19B	9533	11128	7152	27
H1AA	7296	11250	5981	27
H1AB	5703	11061	6289	27
H1C	10568	5924	4162	27
H1DA	7695	12098	8338	45
H1DB	6216	12187	7588	45
H1DC	7792	12400	7272	45

Experimental

The crystal was kept at 100.01(10) K during data collection. Using Olex2 [1], the structure was solved with the Superflip [2] structure solution program using Charge Flipping and refined with the ShelXL [3] refinement package using Least Squares minimisation.

1. Dolomanov, O.V., Bourhis, L.J., Gildea, R.J, Howard, J.A.K. & Puschmann, H. (2009), J. Appl. Cryst. 42, 339-341.

2. Palatinus, L. & Chapuis, G. (2007). J. Appl. Cryst., 40, 786-790; Palatinus, L. & van der Lee, A. (2008). J. Appl. Cryst. 41, 975-984; Palatinus, L., Prathapa, S. J. & van Smaalen, S. (2012). J. Appl. Cryst. 45, 575-580.
3. Sheldrick, G.M. (2015). Acta Cryst. C71, 3-8.
